# Mitonuclear genotype remodels the metabolic and microenvironmental landscape of Hürthle cell carcinoma

**DOI:** 10.1126/sciadv.abn9699

**Published:** 2022-06-22

**Authors:** Ian Ganly, Eric Minwei Liu, Fengshen Kuo, Vladimir Makarov, Yiyu Dong, Jinsung Park, Yongxing Gong, Alexander N. Gorelick, Jeffrey A Knauf, Elisa Benedetti, Jacqueline Tait-Mulder, Luc G.T. Morris, James A. Fagin, Andrew M Intlekofer, Jan Krumsiek, Payam A. Gammage, Ronald Ghossein, Bin Xu, Timothy A. Chan, Ed Reznik

**Affiliations:** 1 Human Oncology and Pathology Program, Memorial Sloan Kettering Cancer Center, New York, NY, USA.; 2 Department of Surgery, Memorial Sloan Kettering Cancer Center, New York, NY, USA.; 3 Computational Oncology Service, Memorial Sloan Kettering Cancer Center, New York, NY, USA.; 4 Lerner Research Institute, Cleveland Clinic, Cleveland, OH, USA.; 5 Marie-Josée and Henry R. Kravis Center for Molecular Oncology, Memorial Sloan Kettering Cancer Center, New York, NY, USA.; 6 Department of Physiology and Biophysics, Weill Cornell Medicine, New York, NY, USA.; 7 Englander Institute for Precision Medicine, Weill Cornell Medicine, New York, NY, USA.; 8 CRUK Beatson Institute, Glasgow, UK.; 9 Department of Medicine, Memorial Sloan Kettering Cancer Center, New York, NY, USA.; 10 Institute of Cancer Sciences, University of Glasgow, Glasgow, UK.; 11 Department of Pathology, Memorial Sloan Kettering Cancer Center, New York, NY, USA.; 12 Center for Molecular Oncology, Memorial Sloan Kettering Cancer Center, New York, NY, USA.

## Abstract

Hürthle cell carcinomas (HCCs) display two exceptional genotypes: near-homoplasmic mutation of mitochondrial DNA (mtDNA) and genome-wide loss of heterozygosity (gLOH). To understand the phenotypic consequences of these genetic alterations, we analyzed genomic, metabolomic, and immunophenotypic data of HCC and other thyroid cancers. Both mtDNA mutations and profound depletion of citrate pools are common in HCC and other thyroid malignancies, suggesting that thyroid cancers are broadly equipped to survive tricarboxylic acid cycle impairment, whereas metabolites in the reduced form of NADH-dependent lysine degradation pathway were elevated exclusively in HCC. The presence of gLOH was not associated with metabolic phenotypes but rather with reduced immune infiltration, indicating that gLOH confers a selective advantage partially through immunosuppression. Unsupervised multimodal clustering revealed four clusters of HCC with distinct clinical, metabolomic, and microenvironmental phenotypes but overlapping genotypes. These findings chart the metabolic and microenvironmental landscape of HCC and shed light on the interaction between genotype, metabolism, and the microenvironment in cancer.

## Teaser

Profound disruption to mitochondrial and nuclear genomes remodels the metabolism and microenvironment of Hürthle cell carcinomas.

## INTRODUCTION

Although most of the recurrent driver mutations in cancer have likely been identified, their impact on tumor phenotypes remains to be fully understood (*1*). One approach to studying the function of driver events is through analysis of cancers with unique, extreme, or pathognomonic genotypes (e.g., ultramutation in the context of *POLE* deficiency in endometrial cancer and loss-of-function mutations in Accessory Protein 1 (*ATP6AP1*) or *ATPase H+* Transporting Accessory Protein 2 (ATP6AP2) in granular cell tumors) that are likely to evoke distinctive molecular and physiological phenotypes (*2*). Because extreme driver alterations are likely to both confer a selective advantage and provoke cellular stress, the phenotypes of tumors bearing these alterations illustrate how cells cope with and seize upon highly disruptive changes to their genome to produce malignancy.

Hürthle cell carcinoma (HCC) is a rare malignant subtype of thyroid cancer characterized by cells with an accumulation of dysfunctional mitochondria (*3*–*6*). Although HCC only accounts for 2 to 5% of all thyroid cancer diagnoses, it is distinguished by a comparatively poor prognosis. The more aggressive form, widely invasive HCC (HWIDE), has a high fatality rate among thyroid cancers, second only to anaplastic thyroid cancer. The standard of care for HCC involves surgical excision (total thyroidectomy with the removal of regional metastases). HCC tumors are nearly always radioactive iodine refractory, and there is currently no known effective chemotherapeutic agent for treating patients with systemic disease. However, mechanistic target of rapamycin kinase (mTOR) inhibitors are currently being clinically evaluated.

We and others recently described the genomic landscape of HCC tumors, which display two exceptional genotypes: mitochondrial DNA (mtDNA) mutations and widespread (near genome-wide) chromosomal loss of heterozygosity (gLOH). While similar somatic alterations arise in other malignancies, they are particularly extreme in HCC: mtDNA mutations are enriched for truncating variants and frequently reach near homoplasmy (variant allele frequencies approaching 100%, thereby affecting nearly every mtDNA in the cell), whereas in most cancers, they arise heteroplasmically, affecting only a fraction of the mtDNA pool (*7*). Consistent with the notion that mtDNA mutations impair mitochondrial respiration, HCC tumors demonstrate intense uptake of F-18 fluorodeoxyglucose (FDG) by positron emission tomography (PET) imaging (*8*–*10*). However, how HCC cells cope with a near-complete impairment of mitochondrial respiration while preserving tumor fitness is not known. In parallel to mtDNA mutations, HCC tumors often display gLOH of numerous chromosomes, which can be accompanied by reduplication of the remaining allele to produce a near-homozygous but diploid genome. The presence of gLOH is enriched in clinically aggressive HCC and is associated with a poor prognosis (*4*). Metastatic chromophobe renal cell carcinomas also display both abundant mtDNA mutations and gLOH of numerous chromosomes, suggesting that mtDNA and gLOH are evolutionarily coselected in multiple tissue lineages (*11*–*13*). However, while gLOH is associated with clinical aggressiveness in HCC, the selective advantage conferred by gLOH is not understood.

Because of the rarity of HCC, molecular analyses have been limited, and although it is widely appreciated that metabolic adaptations play a central role in HCC pathology, there is a poor understanding of the characteristic metabolic and microenvironmental adaptations associated with tumorigenesis and clinically aggressive disease. Here, through a combination of metabolomic, transcriptomic, and immunophenotypic profiling, we describe the metabolic and microenvironmental landscape of HCC tumors. We find that profound disruption of mitochondrial metabolism, including mtDNA mutations and depletion of citrate pools, is common to many forms of thyroid cancer, but those specific metabolic pathways [such as the reduced form of nicotinamide adenine dinucleotide (NADH)–dependent lysine degradation pathway] are specifically disrupted in HCC. In parallel, we find that gLOH is primarily associated with low immune infiltration, suggesting that it confers a selective advantage through the remodeling of the microenvironment. These findings reveal key interactions between exceptional genotypes and molecular phenotypes in HCC and nominate previously unidentified therapeutic targets for a rare disease with urgent unmet clinical needs.

## RESULTS

### HCCs exhibit profound metabolic adaptations consistent with respiratory dysfunction

To study the metabolism of HCC tumors, we completed semiquantitative liquid chromatography–mass spectrometry (LC-MS) metabolomic profiling on 32 HCC primary tumors, including 8 HWIDE tumors that developed recurrence, 11 HWIDE tumors with no recurrence after surgery, 13 minimally invasive HCC (HMIN) tumors, and 16 adjacent normal thyroid specimens. We additionally profiled several non-HCC thyroid neoplasms for exploratory purposes, including five Hürthle adenomas (HAs), four tall cell variants of papillary thyroid carcinomas (TCV-PTCs), and seven poorly differentiated thyroid carcinomas (PDTCs) (table S1A). In total, we measured 728 metabolites (including 324 lipid species and 404 nonlipid species). When we correlated the frozen time of our metabolomics samples to the metabolite abundance, we identified 12 metabolites that were significantly correlated to freezing time (table S1, B and C). Removing these 12 metabolites did not affect any subsequent analysis results, and so we have flagged these metabolites as potentially confounded and left the data intact for others to analyze in the future (table S1C). Most of the HCC primary tumors were previously profiled by RNA sequencing (RNA-seq), which we integrated into the downstream analysis (*4*). Comparing all tumors regardless of histology to normal tissue, we identified 470 total metabolites as differentially abundant (*q* < 0.05, Wilcoxon rank sum test; see Methods and table S1D).

**Fig. 1.**
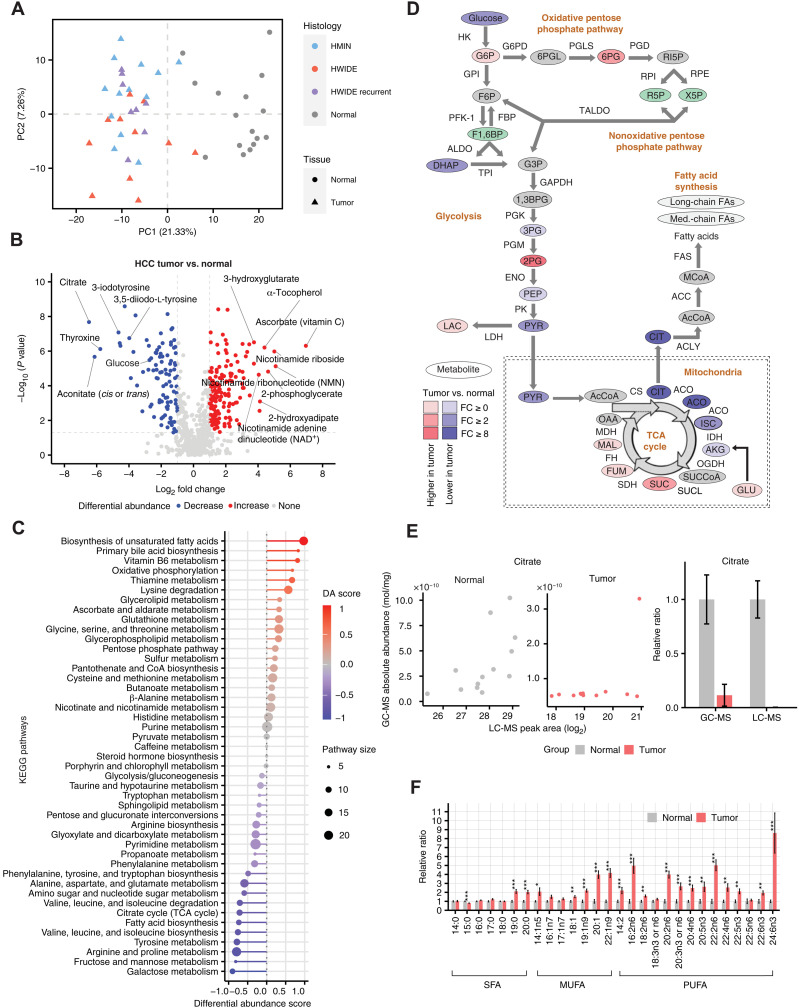
Metabolomic profiling of HCC tumors. (**A**) Tumor and normal samples in the first two components of PCA space. (**B**) Differential metabolite abundance test between HCC tumor and adjacent normal samples. (**C**) DA score shows enriched and depleted KEGG metabolic pathways between HCC tumor and adjacent normal samples. (**D**) Metabolic changes of central carbon metabolism in HCC. Metabolites are labeled as ovals. Enzymes for individual chemical reactions are labeled next to the arrows connecting two metabolites. Color corresponds to the fold changes (FC) between tumor and normal tissues. Red, increase; blue, decrease; green, isomers; gray, not measured. (**E**) Citrate abundance association between gas chromatography followed by mass spectrometry (GC-MS) and LC-MS. (**F**) Relative lipid abundance between HCC tumor and adjacent normal samples (*P < 0.05, **P < 0.01, and ***P < 0.001), stratifying by saturated fatty acids (SFAs), monounsaturated fatty acids (MUFAs), and PUFAs. G6P, glucose-6-phosphate; F6P, fructose 6-phosphate; F1,6BP, fructose 1,6-bisphosphate; DHAP, dihydroxyacetone phosphate; G3P, glyceraldehyde 3-phosphate; 1,3BPG, 1,3-bisphosphoglycerate; 3PG, 3-phosphoglycerate; PEP, phosphoenolpyruvate; PYR, pyruvate; LAC, lactate; 3PHP, 3-phosphohydroxypyruvate; 3PSER, 3-phosphoserine; SER, serine; AcCoA, acetyl-CoA; ISC, isocitrate; CIT, citrate; ACO, *cis*-aconitate; AKG, α-ketoglutarate; SUCCoA, succinyl-CoA; SUC, succinate; FUM, fumarate; MAL, malate; GLU, glutamate; CYS, cysteine; GLY, glycine; MCoA, malonyl-CoA; HCYS, homocysteine; MET, methionine; 6PGL, 6-phosphogluconolactone; 6PG, 6-phosphogluconate; R5P, ribulose 5-phosphate; X5P, xylulose 5-phosphate; GAPDH, glyceraldehyde-3-phosphate dehydrogenase; LDH, lactate dehydrogenase; HK, hexokinase; G6PD, glucose-6-phosphate dehydrogenase; GPI, glucose-6-phosphate isomerase; PFK-1, phosphofructokinase-1; FBP, fructose-1,6-bis-phospharase; ALDO, aldolase; TPI, triosephosphate isomerase; PGLS, 6-phosphogluconolactonase; PGD, phosphogluconate dehydrogenase; RPI, ribose-5-phosphate isomerase; RPE, ribulose 5-phosphate 3-epimerase; TALDO, transaldolase; PGK, phosphoglycerate kinase; PGM, phosphoglucomutase; ENO, enolase; PK, pyruvate kinase; FAS, fatty acid synthase; ACC, acetyl-CoA carboxylase; ACLY, ATP citrate lyase; IDH, isocitrate dehydrogenase; OGDH, oxoglutarate dehydrogenase; SUCL, succinyl-CoA ligase; SDH, succinate dehydrogenase; FH, fumarase; MDH, malate dehydrogenase; RI5P, ribulose-5-phosphate; OAA, oxaloacetate.

Focusing initially on HCC and matched normal tissues, HCC tumors were distinguishable from normal thyroid specimens based on principal components analysis (PCA) (Fig. 1A). When comparing HCC tumors to adjacent normal thyroid tissue, 54% of metabolites (393 of 728 metabolites) were differentially abundant (*q* < 0.05, Wilcoxon rank sum test; Fig. 1B). When comparing clinical subgroups of HCC (HWIDE, associated with four or more foci of vascular invasion; and HMIN, characterized by capsular invasion and/or less than four foci of vascular invasion), we identified zero metabolites with statistically significant changes in abundance at a significant *q*-value threshold of 0.05 (table S1E). Last, to determine whether specific metabolic pathways were particularly affected, we calculated an effect size–weighted differential abundance (DA) score per Kyoto Encyclopedia of Genes and Genomes (KEGG) metabolic pathway (see Fig. 1C and Methods). Among all pathways, the polyunsaturated fatty acid (PUFA) biosynthesis pathway demonstrated the largest DA score and was prioritized for downstream analysis.

Among the largest metabolic changes evident in HCC were the depletion of several thyroid hormone precursors. The thyroid is an endocrine organ responsible for the production of the thyroid hormones 3,3′,5-triiodo-l-thyronine (T_3_), and 3,5,3′,5′-tetraiodo-l-thyronine or thyroxine (T_4_) through the progressive iodination of specific tyrosine residues on the thyroglobulin protein. Three precursors in thyroid hormone biosynthesis were depleted >8-fold in HCC tumors relative to normal thyroid tissues: 3,5-diiodo-l-tyrosine, 3-iodotyrosine, and T_4_ (Fig. 1B), indicating that the production of thyroid hormones is blunted in tumor cells. Consistent with this, in parallel, RNA-seq of 28 overlapping tumor samples, we observed down-regulation of thyroglobulin (encoded by the *TG* gene; *q* = 0.03; fig. S1A) and down-regulation of the sodium/iodide symporter (encoded by the *SLC5A5* gene; *q* = 1.40 × 10^−4^; fig. S1A). Patients with thyroid cancer do not typically display changes in T_3_ and T_4_ serum levels, indicating that the remaining normal thyroid cells are sufficient to maintain thyroid hormone biosynthesis.

Unlike other types of differentiated thyroid carcinomas such as papillary thyroid cancer (PTC) and follicular thyroid cancer, HCC tumors consistently demonstrate high FDG avidity on PET imaging, indicating that they rapidly take up glucose from the tumor microenvironment (TME) (*8*–*10*). Consistent with high heteroplasmy truncating mtDNA mutations primarily affecting complex I in HCC, we observed a significant decrease in the pyruvate/lactate ratio (often used as a surrogate for NAD^+^/NADH) in HCC tumors relative to normal tissues (fig. S1B). Reasoning that HCC likely relies on aerobic glycolysis to generate lactate as a means for glucose catabolism, we therefore investigated metabolomic changes in the glycolysis and tricarboxylic acid (TCA) cycle pathways (Fig. 1D). Free (unphosphorylated) glucose was substantially depleted in tumors (log_2_ fold change = −2.77, *q* = 5.37 × 10^−6^), possibly reflecting a large increase in uptake and phosphorylation by tumor cells. While most glycolytic intermediates demonstrated no statistically significant change in abundance, we noted the accumulation of 2-phosphoglycerate (2PG) (with unknown physiological consequence) to levels 27-fold higher than in adjacent normal tissue (*q* = 1.85 × 10^−5^; Fig. 1, B and C, and table S1F). Most notably, citrate (log_2_ fold change = −6.48, *q* = 2.10 × 10^−8^) and *cis*-aconitate (log_2_ fold change = −6.13, *q* = 2.14 × 10^−6^) were highly depleted in tumors relative to normal tissues (Fig. 1, B and C, and table S1F). To validate the large drop of TCA cycle intermediates in a subset of HCC tumors, we quantified the absolute concentration of citrate using gas chromatography-mass spectrometry (GC-MS) with a standard curve, confirming that citrate was severely depleted in HCC (Fig. 1E).

A significant fraction of the metabolomics panel profiled lipids, including free fatty acids, acylcarnitines, and other complex molecules. Many functional classes of lipids showed an increase in abundance relative to normal, but the size of this effect depended strongly on the specific class of lipids. We observed a tendency for increased levels of desaturation (i.e., the presence of double bonds in fatty acid chains) in free fatty acids in HCC tumors relative to normals. Specifically, we found elevation of 12 of 14 PUFAs compared to 5 of 7 monounsaturated fatty acids (MUFAs) and only 2 of 6 saturated fatty acids (SFAs), with the effect sizes of PUFAs substantially larger than other fatty acid species (Fig. 1F). Although the functional role of increased desaturation is unclear, a recent report noted that the enzymes involved in the desaturation of highly unsaturated fatty acids (HUFAs) are NADH-dependent and that HUFA biosynthesis in the presence of respiratory impairment may be a mechanism for regeneration of oxidized NAD^+^ from NADH (*14*). However, we did not observe a significant association of PUFA levels with the pyruvate/lactate ratio (as a surrogate for NAD^+^/NADH) (table S1G). We noted that, in parallel to PUFA accumulation, HCC tumors also accumulate high levels of antioxidant metabolites including ascorbate (log_2_ fold change = 6.96, *q* = 4.92 × 10^−7^) and oxidized glutathione (log_2_ fold change = 3.06, *q* = 0.06; reduced glutathione, log_2_ fold change = 2.04, *q* = 0.79) that can counteract the accumulation of reactive oxygen species (ROS). To directly assess whether HCC tumors mount a response to ferroptotic susceptibility, we stained a panel of HCC tumors with *GPX4 (*a key mediator of ferroptotic responses through its ability to reduce lipid hydroperoxides) and observed strong positivity of *GPX4* in tumors but not in normal tissues (fig. S2, A and B). Given that peroxidation of PUFAs by ROS can trigger iron-mediated cell death via ferroptosis, our data imply that HCC tumors may accumulate antioxidant species to counteract elevated susceptibility to ferroptosis (*15*).

We hypothesized that some of the large-effect metabolomic phenotypes we observed in HCC could be ascribed to changes in the expression of nearby enzymes in the metabolic network. To test this, we determined the concordance between metabolic gene expression and bulk metabolomics data at the pathway level using matched RNA-seq data of 28 HCC metabolomics samples. We calculated weighted DA scores (accounting for both effect size and statistical significance) for each KEGG metabolic pathway (see Methods and table S1, H and I). In total, 253 of 728 metabolites and 2376 of 16,853 total genes were successfully mapped onto at least one KEGG metabolic pathway. We observed no positive correlation between metabolite-based and RNA-based DA pathway scores (Spearman’s ρ = −0.23, *P* = 0.13; fig. S1C). This discordant trend between metabolite-based and RNA-based pathway scores was consistent with our prior observations in clear cell renal cell carcinoma, where broad down-regulation of most metabolic genes contrasts with heterogeneous and balanced changes of metabolite levels in the KEGG metabolic pathways (*16*).

More granularly, we investigated whether large, tumor-specific metabolomic changes were associated with changes in the expression of enzymes in the same pathway. These changes occur in other cancers, e.g., *FH-* and *SDH-*deficient renal cell carcinomas, where biallelic deletion of the target enzyme leads to extreme accumulation of the upstream metabolites fumarate and succinate, respectively (*17*). In contrast to the extreme drop in citrate and *cis*-aconitate levels, we found that expression of enzymes in both oxidative phosphorylation and the TCA cycle was uniformly elevated in HCC relative to normal thyroid tissues, including a significant up-regulation of citrate synthase (log_2_ fold change = 1.74, *q* = 2.00 × 10^−4^) (fig. S1D). Similarly, the 27-fold up-regulation of 2PG in glycolysis (Fig. 1D) and the large increase in abundance of numerous metabolites in the lysine degradation pathway were not associated with either mutation or large transcriptional changes in any of the surrounding enzymes (fig. S1E). We observed a similar effect with regard to the increased abundance of PUFAs in HCC; although expression of the master transcription factor *SREBF1* was up-regulated in HCC tumors compared to adjacent normal thyroid (log_2_ fold change = 1.10, *q* = 0.041), expression of *SCD* (which introduces double bonds into stearoyl–coenzyme A (CoA) and palmitoyl-CoA and is believed to be the rate-limiting step in MUFA biosynthesis) was not significantly overexpressed in HCC tumors (log_2_ fold change = 1.87, *q* = 0.18). Therefore, it is likely that these large metabolomic adaptations in HCC arise from distal perturbations (e.g., disruption of mitochondrial complex I or accumulation of mitochondria), rather than from changes to proximal enzymes.

### Comparative metabolomics identifies extreme, HCC-specific disruptions of metabolism in the lysine degradation pathway

**Fig. 2.**
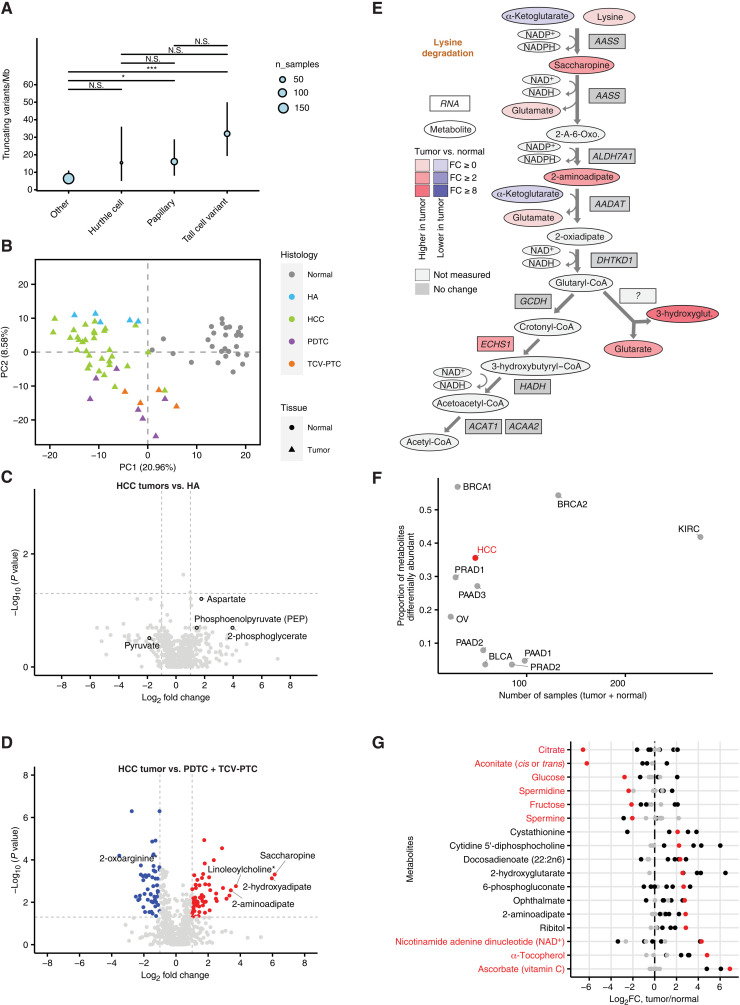
Comparative metabolomics. (**A**) mtDNA mutation burden in different thyroid cancer subtypes. *P < 0.05 and ***P < 0.001. N.S., not significant. (**B**) HCC, HA, PDTC, TCV-PTC, and normal samples in the first two components of PCA space. (**C**) Differential metabolite abundance test between HCC tumor and HA samples. (**D**) Volcano plot of DA test in HCC tumor versus PDTC and TCV-PTC. (**E**) Metabolic changes of lysine degradation pathway in HCC tumors relative to normals. NADP^+^, nicotinamide adenine dinucleotide phosphate; NADPH, reduced form of NADP^+^. (**F**) The proportion of differentially abundant metabolites in tumors relative to normal tissues for HCC and other cancer types. Despite a comparatively small sample size and statistical power to detect changes in metabolite levels relative to datasets, HCC is characterized by a high proportion of differentially abundant metabolites. BRCA1, breast invasive carcinoma, study 1; BRCA2, breast invasive carcinoma, study 2; BLCA, bladder urothelial carcinoma; HCC, hürthle cell carcinoma; KIRC, kidney renal clear cell carcinoma; OV, ovarian serous cystadenocarcinoma; PRAD1, prostate adenocarcinoma, study 1; PRAD2, prostate adenocarcinoma, study 2; PAAD1, pancreatic adenocarcinoma, study 1; PAAD2, pancreatic adenocarcinoma, study 2; PAAD3, pancreatic adenocarcinoma, study 3; and refer to Reznik *et al*. (*22*) for the details in each study. (**G**) Significantly differentially abundant metabolites in HCC (red color) and other cancer types (black color) in (F). Specific metabolites show exceptionally large-magnitude decreases/increases in abundance in HCC, including citrate, aconitate, glucose, NAD^+^, and vitamin C.

An outstanding question in our analysis was the extent to which metabolomic changes in HCC were specific to HCC, compared to other forms of thyroid cancer. Although HCC is characterized by a high burden of mtDNA mutations, prior reports describe mtDNA mutations across numerous thyroid cancer histologies beyond HCC, including conventional PTC (*7*). We reasoned therefore that metabolomic comparisons with other forms of thyroid cancer could potentially distinguish HCC-specific phenotypes from those evident in other (potentially mtDNA-mutated) thyroid cancers. Before doing so, we sought to better assess the mtDNA mutation burden across different thyroid cancer histologies. We therefore analyzed the mtDNA of 309 thyroid cancer samples profiled by our institution’s prospective clinical sequencing platform MSK-IMPACT. We focused on the burden of frameshift insertions/deletions and nonsense mutations, which truncate the protein product, therefore are highly likely to impair function, and which are readily detectable in off-target reads from targeted sequencing data. Unexpectedly, we found that the high burden of truncating mtDNA mutations (20 to 30 mutations/Mb, affecting ~30 to 50% of all tumors) is not unique to HCC tumors but rather is common to several histologies of thyroid cancer (Fig. 2A). Moreover, nearly homoplasmic truncating mtDNA mutations in HCC tumors are also common in PTC and TCV-PTCs (fig. S3A). Of particular interest to us was the high mtDNA mutation rate (and incidence of near-homoplasmic mutations) among tumors classified as TCV-PTC, an FDG-avid variant of conventional PTC known to display oncocytic features that exhibited an mtDNA-truncating mutation rate comparable to, if not, exceeding HCC (Fig. 2A). This suggests that FDG avidity may correlate, if only roughly, with the presence of high-heteroplasmy mtDNA mutations in thyroid cancer.

To understand the specificity of metabolic phenotypes in HCC relative to other types of thyroid cancer, we jointly analyzed metabolomic data of all thyroid cancer samples together. PCA demonstrated that histology-specific effects were dominant, suggesting that each histology has a set of characteristic metabolic features (Fig. 2B). We focused initially on comparing HCC to HAs, which represent a benign oncocytic tumor in the thyroid gland that resembles HCC with respect to the accumulation of dysfunctional mitochondria within HA cells. However, HAs do not show features of malignancy such as capsular invasion and vascular invasion, which occur in HCC. When considering a PCA projection of HA tumors profiled in our metabolomics dataset, HCC and HA tumors fell into distinct but overlapping clusters. Similarly, only 2 of 724 metabolites (hypoxanthine and *N*-acetylthreonine) were differentially abundant (*q* < 0.05, eight metabolites with a relaxed q-value threshold of 0.1; Fig. 2C and table S2A). These small differences in metabolic profile between HCC and HA indicate that the metabolic changes observed in Hürthle cell neoplasms are unlikely to be solely responsible for the differences in biological behavior between the benign HA and the malignant HCC.

The characteristic oncocytic presentation of HCC, with a cytoplasm filled with dysfunctional mitochondria, led us to hypothesize that HCCs are distinguished from other thyroid cancer types by changes to metabolic pathways linked to mitochondrial health and integrity. We therefore metabolomically compared HCC to PDTC and TCV-PTC. In aggregate, 175 of 395 metabolites that were significantly differentially abundant in HCC tumors relative to normal tissue were also similarly differentially abundant when comparing PDTC and TCV-PTC to normal tissue (*q* < 0.05, Wilcoxon rank sum test; fig. S4A and tables S1D and S2B). Several functionally related groups of metabolites demonstrated consistent changes in HCC, TCV-PTC, and PDTC relative to adjacent normal tissues (Fig. 2D, fig. S4B, and table S2C). First, we observed that all thyroid cancers relative to adjacent normal tissues were extremely depleted of thyroid hormone precursors including 3-iodotyrosine, 3,5-diodo-l-tyrosine, and T_4_ (Fig. 2D). Second, we found consistent up-regulation in all profiled subtypes of thyroid cancer of antioxidant metabolites or their derivatives including ascorbate, α-tocopherol, and CoA-glutathione. Third, we noted that derivatives of NAD^+^, including nicotinamide riboside and nicotinamide ribonucleotide, were at significantly higher abundance in all thyroid histologies (Fig. 2D). Last, and of the largest overall magnitude, we observed that all thyroid tumors exhibited consistent and large changes in the abundances of metabolites in central carbon metabolism, including increases in 2PG and large depletions in glucose, citrate, and *cis*-aconitate (Fig. 2D). The depletion of citrate was particularly stark. As a group, HCC, PDTC, and TCV-PTC exhibited a large depletion of both citrate (log_2_ fold change = −4.43, *q* = 3.55 × 10^−13^) and *cis*-aconitate (log_2_ fold change = −3.74, *q* = 1.39 × 10^−9^) relative to normal thyroid tissue (fig. S4C). Together, the above data suggest that HCC, PDTC, and TCV-PTC thyroid tumors, similar to clear cell renal cell carcinomas, preferentially route glucose-derived carbon toward lactate and away from the TCA cycle. Moreover, these data suggest that cells derived from the thyroid lineage are endowed with the special capability of surviving the extreme depletion of TCA cycle intermediates.

Despite the similarities noted above between HCC and non-HCC tumors in central carbon metabolism, numerous metabolic features also distinguished HCC from the non-HCC thyroid cancer histologies. The most prominent of these differences occurred in metabolites associated with the lysine degradation pathway (Fig. 2E). Lysine is an essential amino acid whose degradation occurs via two distinct pathways: a mitochondrially localized saccharopine pathway (in which lysine condenses with α-ketoglutarate to produce saccharopine) and a cytosolically localized pipecolate pathway (*18*). Both pathways converge to produce 2-aminoadipate. Subsequent catabolism of 2-aminoadipate produces glutaryl-CoA, which can be further metabolized through two pathways, producing either acetyl-CoA or, pathologically, 3-hydroxyglutarate and glutarate (Fig. 2E). We observed ~6-fold accumulation of saccharopine (log_2_ fold change = 2.68, *q* = 9.23 × 10^−4^) but no such accumulation of pipecolate (log_2_ fold change = −0.51, *q* = 0.03), indicating that the saccharopine pathway is specifically perturbed. Furthermore, we observed >8-fold accumulation of the intermediates 2-aminoadipate, 3-hydroxyglutarate, and glutarate specifically in HCC, but not in other thyroid cancer histologies (Fig. 2E and fig. S4D). Glutamate, which is not only produced in the metabolism of saccharopine but can also be derived from extracellular glutamine, demonstrated only modest increases in abundance (log_2_ fold change = 0.27, *q* = 0.01). Notably, the accumulation of 3-hydroxyglutarate and glutarate in the urine phenocopies the presentation of glutaric aciduria type I, an autosomal recessive disorder caused by a deficiency in glutaryl-CoA dehydrogenase (GCDH), which catalyzes the conversion of glutaryl-CoA to crotonyl-CoA (*19*–*21*). GCDH is neither underexpressed nor mutated in HCC tumors. GCDH oxidizes glutaryl-CoA and transfers them to the electron transfer flavoprotein, an electron acceptor for a wide spectrum of dehydrogenases that ultimately donates electrons to the ubiquinone pool in the mitochondrial electron transport chain (ETC). Given that GCDH is neither mutated nor transcriptionally down-regulated in HCC, this suggests that the accumulation of metabolites upstream of GCDH and overflow into glutarate/3-hydroxyglutarate may instead be thermodynamically driven by the unfavorability of GCDH in the presence of extreme ETC dysfunction. Evaluation of this hypothesis with isotope-tracing experiments remains difficult because of the scarcity of cell line models for HCC.

The extreme metabolic alterations in central carbon metabolism evident in HCC (and other thyroid cancer types) prompted us to compare metabolomic aspects of HCC to tumors of other tissue lineages. To do so, we leveraged a harmonized pan-cancer metabolomics dataset produced by our group, which contains metabolomic data from six different cancer types using public metabolomics data from 10 studies (*22*). Critically, each original dataset was informatically and quantitatively standardized using a common analytical pipeline, enabling comparisons of tumor-specific metabolic alterations across cancer types. Compared to other cancers, HCC tumors display a relatively high proportion of differentially abundant metabolites in tumors relative to adjacent normal tissues (Fig. 2F). This proportion of metabolomic changes in HCC tumors is similar to clear cell renal cell carcinoma (Fig. 2F). Focusing on the 41 significant differentially abundant metabolites in HCC (*q* < 0.05 and absolute value of log_2_ fold change > 1, Wilcoxon rank sum test) that were measured in at least five different studies, we observed that the depletion of citrate and *cis*-aconitate (and to lesser extent glucose, fructose, and spermidine) was far more extreme in magnitude than in any other cancer type in our dataset. Second, we also noted that a number of metabolites related to the oxidative stress response (ascorbate, α-tocopherol, and the glutathione analog ophthalmate), as well as the lysine degradation intermediate 2-aminoadipate, all demonstrated an exceptionally large elevation in HCC tumors compared to other cancer types (Fig. 2G and fig. S4E). These data indicate that the distinguishing metabolic features of HCC (which are shared to some extent with other thyroid cancers) are a large depletion of citrate and *cis*-aconitate, depletion of glucose, impairment of lysine degradation, and an accumulation of ROS-responsive metabolites.

### The immune landscape of HCC

**Fig. 3.**
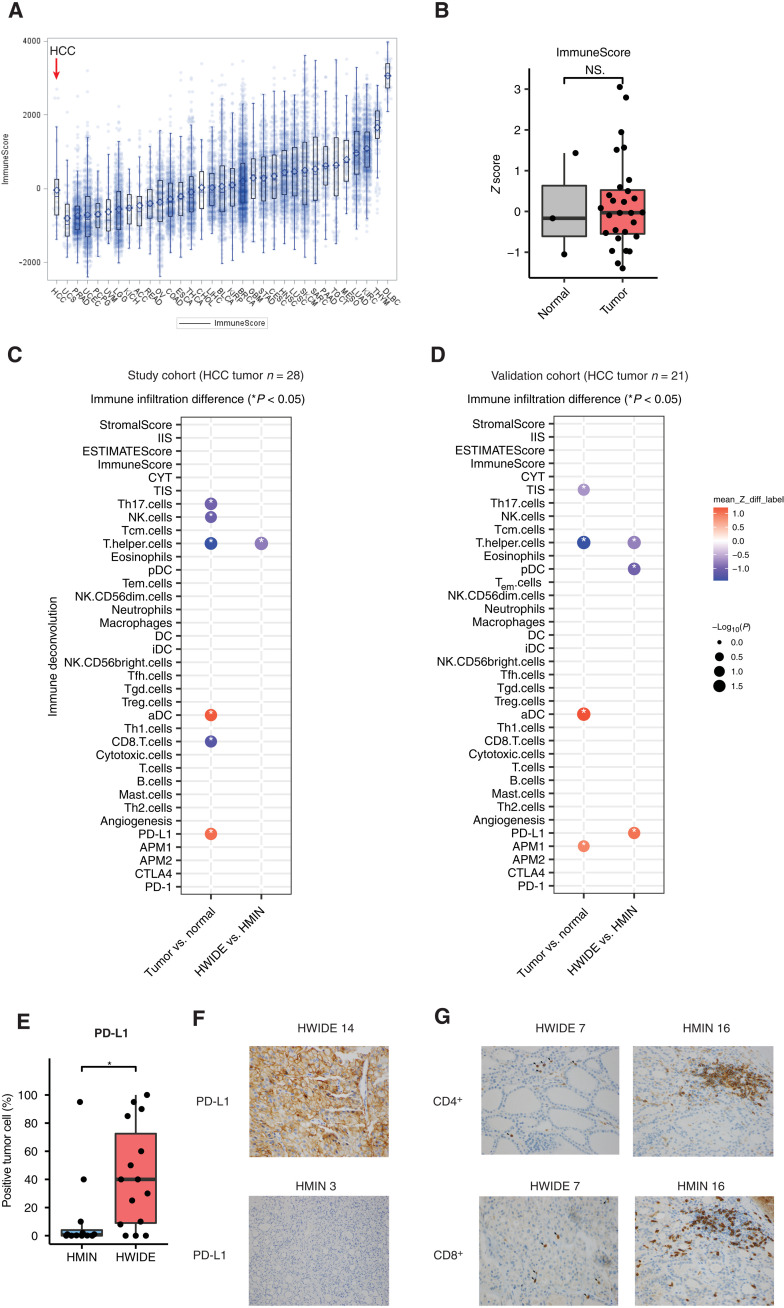
Immune landscape of HCC. (**A**) Overall immune infiltration (ImmuneScore) of HCC and other cancer types in the TCGA. (**B**) HCC tumors have a comparable overall immune infiltration to their adjacent normal samples. iDC, immature dendritic cells; APM1, MHC class I antigen processing machinery; CTLA, cytotoxic T-lymphocyte-associated protein 4. (**C**) Significant TME features discriminating HCC tumor and adjacent normal samples or HWIDE and HMIN in the study cohort (28 HCC tumor samples). (**D**) Significant TME features discriminating HCC tumor and adjacent normal samples or in HWIDE and HMIN in the validation cohort (21 HCC tumor samples). (**E** and **F**) PD-L1 is increased in expression in HWIDE relative to HMIN. *P < 0.05. (**G**) Representative pathology images show the decreased expression of CD4^+^ and CD8^+^ in the HWIDE phenotype compared to the HMIN phenotype.

Because of the rarity of HCC, little is known about the HCC microenvironment. We therefore analyzed the cellular composition of the HCC TME using RNA-seq and immunohistochemical (IHC) approaches. First, we applied several immune deconvolution methods including ESTIMATE, single-sample gene set enrichment analysis (ssGSEA), and the cytolytic activity score to RNA-seq data of 28 HCC tumors profiled with metabolomics in our cohort (*23*–*25*). On the basis of ESTIMATE scores of tumor purity from RNA-seq, HCC tumors ranged from 47 to 99% in purity and were consistent with orthogonal estimates of tumor purity from DNA sequencing of the same tumors using FACETS (fig. S5A) (*26*). Levels of immune infiltration in HCC were comparable to PTC samples profiled in the Cancer Genome Atlas (TCGA) and comparatively lower relative to other solid tumor types (Fig. 3A).

Despite a comparable level of overall immune infiltration (ImmuneScore) in the HCC TME relative to adjacent normal thyroid tissue (Fig. 3B), the abundance of specific cell populations in HCC was distinct from those in the normal thyroid (Fig. 3C and table S3A). Relative to normal tissue, HCC tumors had elevated expression of immune checkpoint markers [e.g., programmed death-ligand 1 (PD-L1), *P* = 2.99 × 10^−2^, Wilcoxon rank sum test] and activated dendritic cells (aDCs; *P* = 2.99 × 10^−2^, Wilcoxon rank sum test) (Fig. 3C and table S3A). In contrast, HCC tumors had comparatively lower infiltration of CD8^+^ cells (CD8.T.cells, *P* = 3.53 × 10^−2^, Wilcoxon rank sum test), T helper 17 cells (Th17.cells, *P* = 2.52 × 10^−2^, Wilcoxon rank sum test), T helper cells (T.helper.cells, *P* = 2.12 × 10^−2^, Wilcoxon rank sum test), and natural killer (NK) cells (NK.cells, *P* = 4.16 × 10^−2^, Wilcoxon rank sum test) (Fig. 3C and table S3A). In an independent cohort of 21 HCC tumors with RNA-seq data, aDCs (*P* = 1.14 × 10^−2^, Wilcoxon rank sum test) and T helper cells (T.helper.cells, *P* = 1.14 × 10^−2^, Wilcoxon rank sum test) were similarly differentially abundant (Fig. 3D and table S3B). To further investigate the microenvironment of HCC, we completed IHC staining of cell markers of interest among 27 HCC tumors with sufficient tissue available (see Methods). Notably, with the exception of a single tumor showing nearly no CD8^+^ infiltration, all other tumors demonstrated CD8^+^ T cells mixed with tumor cells. While most HCC tumors do not have a stromal component, the small fraction that did have fibrotic stroma demonstrated T cells dispersed throughout both the tumor and the stromal regions, indicating that immune-excluded phenotypes (where immune cells infiltrate surrounding stroma but not tumor) are uncommon in HCC. This indicates that although HCC contains a comparable level of immune infiltration to normal thyroid tissue, cytotoxic cells are reduced in the HCC TME in favor of increased levels of antigen-presenting cells.

While HWIDE and HMIN tumors were indistinguishable from the metabolomics data, we found that the HWIDE/HMIN status was a critical determinant of TME composition (Fig. 3C and table S3A). Specifically, while HWIDE and HMIN tumors grossly resembled each other in terms of the extent of immune cell infiltration (ImmuneScore), a more granular analysis revealed that HWIDE tumors had significantly lower T helper cells (T.helper.cells, *P* = 1.13 × 10^−2^, Wilcoxon rank sum test) compared to HMIN tumors (Fig. 3C and table S3A). These effects were corroborated in an independent cohort (T.helper.cells, *P* = 1.24 × 10^−2^, Wilcoxon rank sum test) (Fig. 3D and table S3B). Expression of PD-L1 analyzed by IHC was increased in the HWIDE tumors relative to HMIN (mean HWIDE versus HMIN 40% tumor cells positive versus 12% tumor cells positive, *P* = 0.03). Similarly, IHC analysis indicated that HWIDE tumors were depleted of CD8^+^ and CD4^+^ T cells relative to HMIN, although these results did not reach statistical significance (CD4^+^ T cells: mean HWIDE versus HMIN, 16 positive cells/high-power field (HPF) hotspot versus 52 positive cells/HPF hotspot, *P* = 0.4; CD8^+^ T cells: mean HWIDE versus HMIN, 26 positive cells/HPF hotspot versus 56 positive cells/HPF hotspot, *P* = 0.13; CD68^+^ cells: mean HWIDE versus HMIN, 43 positive cells/HPF hotspot versus 70 positive cells/HPF hotspot, *P* = 0.26, Fig. 3G).

### Integrative analysis of RNA and metabolite data reveals distinct clusters of HCC tumors

**Fig. 4.**
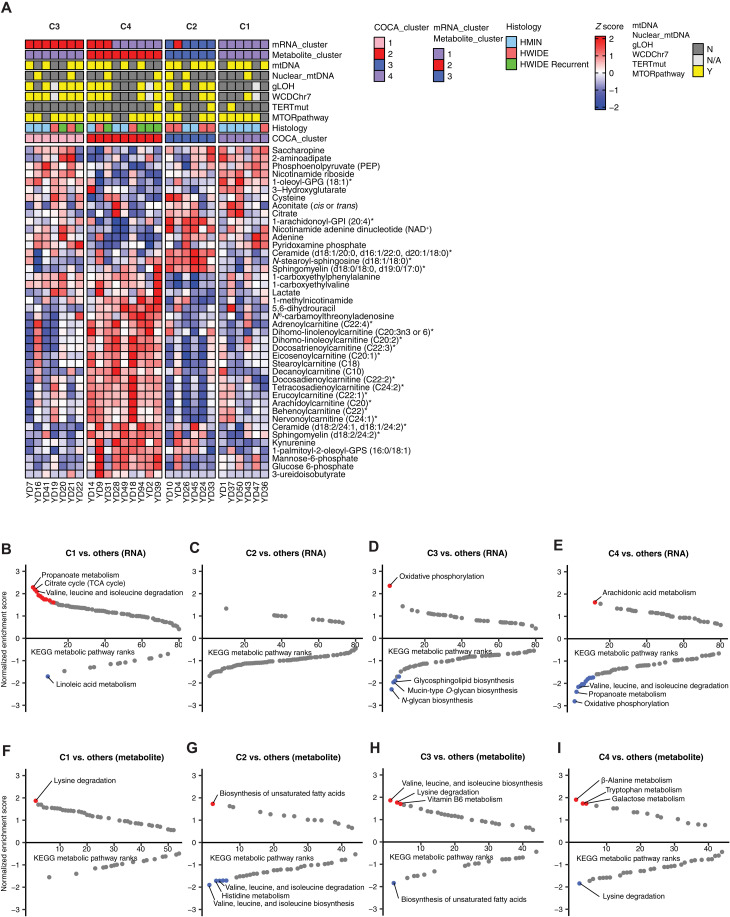
Integrated analysis of metabolomics and gene expression data in HCC. (**A**) Metabolite abundance across COCA clusters. The top two rows (mRNA_cluster and metabolite_cluster) show the clustering membership from a single-data modality, RNA-seq, or metabolomics data. The top third to seven rows show genetic or clinical alteration information. nuclear_mtDNA, mutation in genes in the nuclear DNA related to mitochondrial complex; WCDChr7, whole chromosome duplication at chromosome 7; TERTmut, mutation in TERT promoter; mTORpathway, mutations in the mTOR pathway; histology, histology assignment. (**B** to **E**) Significant enriched (red) or depleted (blue) KEGG metabolic pathways from RNA GSEA analysis in each consensus cluster (only the labels of top three enriched or depleted pathways are shown). (**F** to **I**) Significant enriched (red) or depleted (blue) KEGG metabolic pathways from metabolite GSEA analysis in each consensus cluster (only the labels of top three enriched or depleted pathways are shown).

We reasoned that integrative analysis of the multiple data modalities at hand might reveals distinct molecular subtypes of HCC obscured by analysis of either RNA-seq or metabolomics alone. To investigate this, we first generated clustering assignments from consensus clustering in RNA-seq (*n* = 53) and in metabolomics (*n* = 32) separately. For 28 HCC tumor samples both having RNA-seq and metabolomic data, we encoded the gene expression and metabolomics subtype calls of these 28 samples into a binary matrix as the input for the cluster-of-cluster assignment (COCA) (see Fig. 4A, fig. S6A, and Methods) (*27*, *28*). We assigned the name of each identified cluster in COCA according to their proportion of clinically aggressive tumors (HWIDE and recurrent HWIDE), ranging from cluster C1 (least proportion of HWIDE and recurrent HWIDE) to cluster C4 (the highest proportion of HWIDE and recurrent HWIDE) (Fig. 4A). HMIN and HWIDE tumors were found in all four clusters, suggesting that the invasiveness of HCC is not, on its own, sufficient to molecularly stratify tumors. Instead, tumor stratification was qualitatively associated with the interaction of several genomic features (mtDNA mutations, mTOR pathway activation, and LOH from uniparental disomy) and pathology subtypes (HWIDE/HMIN status). For example, cluster C1 is characterized by HMIN tumors with mTOR pathway activation but no gLOH, whereas cluster C3 is characterized by those with gLOH and mTOR activation; in contrast, HMIN tumors in clusters C2 and C4 predominantly had neither gLOH nor mTOR pathway activation (Fig. 4A). The most aggressive tumors, recurrent HWIDE, were found exclusively in the clusters C3 and C4 and (as described by us and others in earlier reports) were characterized by elevated rates of mTOR pathway activation, gLOH, and chromosome 7 amplification.

To directly investigate the contribution of genetic mutations/copy number aberrations to metabolic phenotype, we asked whether metabolite or transcript levels were differentially expressed in the presence/absence of the four most common somatic genotypes described in previous genomic analysis of HCC: mtDNA mutations, gLOH, telomerase reverse transcriptase (*TERT*) mutations, and mTOR pathway alterations (Table 1). Unexpectedly, with the exception of the depletion of spermine in HCC tumor samples with gLOH (log_2_ fold change = −3.69, *q* = 6.68 × 10^−6^), we observed no statistically significant association between individual genomic feature and metabolite levels (fig. S6, B to E). We interpret these findings to mean that metabolite levels are likely influenced by combinations of molecular alterations and changes in the microenvironment rather than individual events. To investigate whether these clusters were associated with different mortality or response to intervention, we conducted Kaplan-Meier analysis on recurrence-free and overall survival (fig. S7, A and B, and table S4A). While not statistically significant due to under power, these data are consistent with our conclusion that patients in the C3 and C4 groups are at elevated risk for metastasis and death.

**Table 1.** Summary of integrated molecular characteristics of HCC. Summary of integrated landscape from genetic, transcriptomic and metabolomics platforms in HCC tumors.

	**C1**	**C2**	**C3**	**C4**
**Samples**	6/28 (21%)	6/28 (21%)	7/28 (25%)	9/28 (32%)
**HMIN**	5/6 (83%)	2/6 (33%)	3/7 (43%)	3/9 (33%)
**HWIDE**	1/6 (17%)	4/6 (67%)	4/7 (57%)	6/9 (67%)
**Recurrence**	0/6 (0%)	0/6 (0%)	2/7 (29%)	4/9 (44%)
**mTOR pathway**	5/6 (83%)	4/6 (67%)	6/7 (86%)	7/9 (78%)
**mtDNA**	4/6 (67%)	5/6 (83%)	4/7 (57%)	5/9 (56%)
**LOH/UPD**	0/6 (0%)	4/6 (67%)	5/7 (71%)	4/9 (44%)
**Gene expression pathway**	Upregulation of genes in the TCA cycle	Upregulation of MYC target pathway, downregulation of genes in the TCA cycle	Upregulation of oxidative phosphorylation	Upregulation of inflammation and the immune response
**Metabolic pathway**	Enriched lysine degradation	Elevation of ceramide/ sphingomyelin species	Enriched valine, leucine and isoleucine biosynthesis, and lysine degradation	Elevation of acylcarnitine species, kynurenine, and 1-methylnicotinamide, depletion of saccharopine and 3 constituents of the NAD^+^ pathway
**TME signatures**	Depletion of CD8^+^ T cells and elevation of PDL1	Depletion of Th cells and elevation of PDL1 and other immune checkpoints	Depletion of Th cells	Enriched regulatory T cells, exhausted T cells and depletion of Th17 cells
**Gene expression**			Upregulation of *NEAT1, MALAT1*	Upregulation of *IDO1,* chemokine ligand family members

Abbreviations: HCC, Hürthle cell carcinoma; C, cluster; HMIN, minimally invasive HCC; HWIDE, widely invasive HCC; mTOR, mechanistic target of rapamycin; mtDNA, mitochondrial DNA; LOH/UPD, loss of heterozygosity/uniparental disomy; MYC, MYC proto-oncogene, bHLH transcription factor; TCA, citric acid cycle; NAD, nicotinamide adenine dinucleotide; TME, tumor microenvironment; CD8, cluster of differentiation 8; PDL1, programmed death-ligand 1; Th, T helper

From pathway-level GSEA, each cluster exhibited distinct transcriptomic and metabolic features (Fig. 4, B to I, Table 1, and table S4, B to I), including marked elevation of lysine degradation metabolites in C1, PUFAs (C2), and expression of long noncoding RNAs *MALAT1 and NEAT1* (C3). Compared to the other three clusters, C4 demonstrated the most molecularly distinct phenotype and also represented the most biologically aggressive cluster with four of six tumors demonstrating recurrence (Fig. 4, A, E, and I, and table S4, E and I). C4 tumors displayed widespread elevation of acylcarnitine species, as well as the immunomodulatory metabolites as kynurenine and 1-methylnicotinamide and concomitant depletion of saccharopine and three constituents of the NAD^+^ pathway (Fig. 4A). From a transcriptomics standpoint, C4 tumors demonstrated an up-regulation of a number of genes related to inflammation and the immune response, including several chemokine (C-X-C motif) ligand family members and the kynurenine-producing enzyme *IDO1* (fig. S6A). C4 tumors also demonstrated elevated macrophage M1 and M2 signatures related to HCC tumors in other COCA cluster (fig. S7, C and D). Together, these data argue that HCC tumors can be molecularly subclassified into four groups with distinct metabolomic and transcriptomic features that partially segregate alongside clinical phenotypes.

**Fig. 5.**
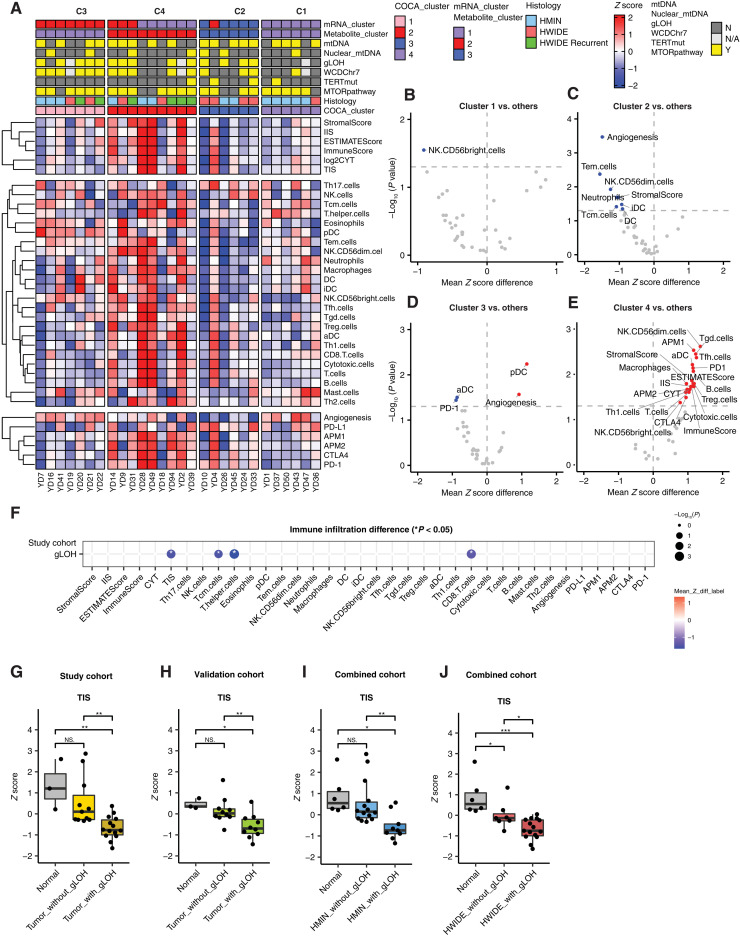
Integrated analysis of TME signatures in HCC. (**A**) TME signatures in each COCA cluster. (**B** to **E**) Significant TME features in each COCA cluster versus others. (**F**) Significant TME features in HCC tumor with gLOH versus without gLOH in the study cohort (28 HCC tumor samples). (**G**) HCC tumors with gLOH have lower T cell infiltration score (TIS) than either HCC tumors without gLOH or normal samples in the study cohort. (**H**) HCC tumors with gLOH have lower TIS than either HCC tumors without gLOH or normal samples in the validation cohort. (**I**) HMIN tumors with gLOH have lower TIS than either HMIN tumors without gLOH or normal samples in the combined cohort. (**J**) HWIDE tumors with gLOH have lower TIS than either HWIDE tumors without gLOH or normal samples in the combined cohort. *P < 0.05, **P < 0.01, and ***P < 0.001.

The association between C4 and both immunomodulatory metabolites and immune-related gene expression programs led us to investigate the variation of TME composition relative to other clusters (Fig. 5, A to E, and table S5, A to D). This revealed that each cluster was associated with at least one unique TME feature. For example, C1: depletion of NK CD56 bright cells (NK.CD56bright.cells, *P* = 2.83 × 10^−2^, Wilcoxon rank sum test); C2: depletion of angiogenic gene expression (*P* = 3.40 × 10^−4^, Wilcoxon rank sum test); C3: depletion of aDCs and PD-1 (aDC, *P* = 3.15 × 10^−2^; PD-1, *P* = 3.64 × 10^−2^, Wilcoxon rank sum test) and elevation of plasmacytoid DC and angiogenesis signature (pDC, *P* = 5.77 × 10^−3^; angiogenesis, *P* = 2.71 × 10^−2^, Wilcoxon rank sum test). Of particular interest to us was C4, which demonstrated the highest level of immune infiltration (ImmuneScore). The increase in immune cell infiltration was largely driven by immunosuppressive cell populations, including regulatory T cells (T_reg_.cells, *P* = 1.61 × 10^−2^, Wilcoxon rank sum test) (Fig. 5E and table S5D). We also have stained HCC tumors with *FOXP3* marker and observed elevated *FOXP3* in C4 related to tumors in other clusters, although because of the small sample size, this did not reach statistical significance (*P* = 0.21, Wilcoxon rank sum test; fig. S7E). C4 tumors also had elevated levels of kynurenine (log_2_ fold change = 2.61, *q* = 3.02 × 10^−2^, Wilcoxon rank sum test) that has been described to promote the differentiation of T cells to a regulatory identity, suggesting that the accumulation of kynurenine and potentially other metabolites may actively shape the cellular composition of the TME (Figs. 4A and 5A). These data indicate that HWIDE tumors adopt at least two fundamentally distinct TME phenotypes: one characterized by immune infiltration comparable to normal phenotype (C2/C3 tumors) and a second characterized by an immune-suppressed phenotype (C4 tumors).

We noted that the distinguishing genomic feature of immune-low C2 and C3 tumors was gLOH (9 of 13 in C2 and C3 tumors and 5 of 15 in all other tumors; *P* = 0.07, chi-squared test). Although the functional consequence of gLOH is not well understood in HCC, we reasoned that this genotype may predispose tumors to adopt a specific TME phenotype. To test this hypothesis, we compared the TME composition of HCC tumors with and without gLOH (Fig. 5F). Tumors with gLOH had a significantly lower T cell infiltration score (TIS; *P* = 2.38 × 10^−3^, Wilcoxon rank sum test), as well as lower levels of CD8^+^ T cells (CD8.T.cells; *P* = 1.12 × 10^−3^, Wilcoxon rank sum test) and other immune cell populations (Fig. 5F). These findings were largely corroborated in an independent set of 21 HCC tumor samples with RNA-seq that did not have matched metabolomics data (TIS, *P* = 7.28 × 10^−3^, Wilcoxon rank sum test) (Fig. 5, G and H). Notably, while gLOH is common in HWIDE tumors (67%, 10 of 15 HWIDE tumors with gLOH), it also appears in HMIN tumors at a lower but still substantial frequency (31%, 4 of 13 HMIN tumors with gLOH). Considering only HMIN tumors, we again observed that the presence of gLOH is associated with lower levels of T cells than non-gLOH HMIN tumors (TIS, *P* = 7.27 × 10^−2^, Wilcoxon rank sum test) (Fig. 5I). Together, these findings suggest that gLOH in HCC results in a microenvironment with reduced immune infiltration that potentially marks a distinct class of HCC tumors and resembles the immunosuppression phenotype associated with tumors with extensive aneuploidy (*29*). Together, these data imply that gLOH confers a selective advantage to HCC tumors in part through immune evasion.

## DISCUSSION

Here, we have described the characteristic, metabolomic, and microenvironmental features of HCC, as well as their relation to two prolific alterations to the HCC genome, high-heteroplasmy mtDNA mutations, and extensive loss of heterozygosity. Our analysis highlights the profound metabolic reorganization of mitochondrial metabolism in many thyroid cancers and the characteristic changes to specific metabolic pathways unique to HCC. We find that while HCC is not strongly immune-depleted relative to normal thyroid tissue, it undergoes a remodeling of the TME that is strongly associated with the presence of gLOH.

The functional consequences of somatic mtDNA mutations in tumors, as well as the selective advantage they may confer, remain a point of intense debate (*30*). The mitochondrial genome encodes 13 proteins and associated ribosomal RNA and tRNA molecules essential for mitochondrial respiration. Data from our group recently demonstrated that pathogenic mtDNA mutations disrupting the reading frame of these 13 protein-coding genes are common across cancer types and affect approximately 1 in 10 cancers regardless of tissue of origin. However, the vast majority of these truncating mutations are heteroplasmic, suggesting that tumor cells retain a pool of wild-type mtDNA that could be used to maintain a minimal respiratory capacity. What distinguishes HCC (and apparently other thyroid cancer types, see Fig. 2A) from other cancers with truncating mtDNA mutations is the presence of near homoplasmy, rendering some of these tumors apparently devoid of wild-type mtDNA. This observation suggests that the metabolic physiology of follicular thyroid cells is able to tolerate severe disruption of respiration (especially of complex I) and the ensuing consequences on the TCA cycle and peripheral metabolism. A tenuous analogy can be drawn to renal cell carcinomas, which similarly acquire high burdens of truncating mutations to complex I and biallelic alterations to the TCA cycle enzymes *SDHB* and *FH* (*31*). However, unlike renal cell carcinoma, thyroid cancers undergo high-magnitude drops in the TCA cycle intermediates citrate and *cis*-aconitate, suggesting that the mechanism for compensation is distinct in the thyroid and the kidney. That HCC can proliferate malignantly in the context of such potent disruption of respiration suggests that its viability is now independent of an intact ETC and motivates a new therapeutic strategy that targets pathways that are synthetically lethal with respiratory incompetency.

Current research on the development of novel treatments for HCC is focused on mTOR inhibitor therapy either alone or in combination with tyrosine kinase inhibitors. A recent phase 2 trial using everolimus in combination with sorafenib has shown promising results (*32*). Our multimodal clustering of the metabolome and the transcriptome reveals that HCC tumors fall into molecularly distinct subgroups that transcend both clinical and genomic categorization and potentially nominate new metabolic and immune-based therapeutic strategies (Table 1). For example, a significant fraction of TME variation can be ascribed to the presence or absence of gLOH, a feature that is associated with invasiveness and recurrence in HCC. These findings suggest that one mechanism through which gLOH promotes aggressive disease is through suppression of cytotoxic immune responses and nominates therapies that reinvigorate the immune system as a potential new treatment modality in HCC tumors with gLOH. From a metabolic perspective, drugs that target lysine degradation, as well as therapies that seize on the vulnerability to ferroptosis of cells laden with high levels of PUFAs, also warrant further investigation in HCC. Accumulation of lysine degradation intermediates may also produce peripheral effects, e.g., by promoting increased posttranslational glutarylation of histones, that may itself produce a targetable epigenetic phenotype. Combining metabolic therapies with drugs that can modulate the TME in genomically defined patients may, therefore, represent a promising new avenue for translational investigation in HCC (*33*).

## METHODS

### Tumor samples

Tumor and matched nonneoplastic normal tissue specimens were obtained from 40 patients with HCC. All tissue samples were snap-frozen in liquid nitrogen at the time of surgery and stored at −80°C. Hematoxylin and eosin–stained tumor sections were reevaluated by a head and neck pathologist (R.G.), confirming the diagnosis of HCC and the classification into either minimally invasive HCC (HMIN) or widely invasive HCC (HWIDE). We detail our exact definition of minimally and widely invasive as follows: HMIN was defined as encapsulated tumor harboring <4 foci of vascular invasion (foci of vascular invasion that were closely adjacent to one another were counted as separate foci) and lacking both gross invasions and vascular invasion of extrathyroid vessels. HWIDE was defined as a tumor with gross invasion/significant vascular invasion if the tumor was grossly invasive, had extrathyroid vascular invasion, and/or was encapsulated with four or more foci of vascular invasion. The terms HMIN and HWIDE are abbreviations specific to our study to define “minimally invasive HCC” and “widely invasive HCC.” All patients were consented to an institutional tissue banking protocol for secondary analysis.

### DNA sequencing, RNA-seq, and variant calling

DNA and RNA-seq data were previously obtained and described in (*4*).

### Metabolomic profiling

Metabolomics profiling of 49 primary tumors and 27 adjacent-normal tissue samples in HCC, HA, PDTC, and TCV-PTC was conducted with Metabolon Inc. using methods detailed below.

### Metabolomics sample preparation

Several recovery standards were added before the first step in the extraction process for quality control. To remove protein, dissociate small molecules was bound to protein or trapped in the precipitated protein matrix, and to recover chemically diverse metabolites, proteins were precipitated with methanol under vigorous shaking for 2 min using a Glen Mills GenoGrinder 2000 and subsequently centrifuged. The resulting extract was divided into five fractions: two for analysis by two separate reverse-phase (RP)/ultrahigh-performance LC–MS/MS (UPLC-MS/MS) methods with positive ion mode electrospray ionization (ESI), one for analysis by RP/UPLC-MS/MS with negative ion mode ESI, one for analysis by hydrophilic interaction liquid chromatography (HILIC)/UPLC-MS/MS with negative ion mode ESI, and one sample was reserved for backup. Samples were placed briefly on a TurboVap (Zymark) to remove organic solvent. The sample extracts were stored overnight under nitrogen before analysis.

### Ultrahigh-performance liquid chromatography–tandem mass spectroscopy

All methods used a Waters ACQUITY UPLC and a Thermo Fisher Scientific Q-Exactive high-resolution/accurate mass spectrometer interfaced with a heated ESI (HESI-II) source and Orbitrap mass analyzer operated at 35,000 mass resolution. The sample extract was dried then reconstituted in solvents compatible to each of the four methods listed above. Each reconstitution solvent contained a series of standards at fixed concentrations to ensure injection and chromatographic consistency. One aliquot was analyzed using acidic positive ion conditions, chromatographically optimized for more hydrophilic compounds. In this method, the extract was gradient-eluted from a C18 column (Waters UPLC BEH C18, 2.1 mm × 100 mm, 1.7 μm) using water and methanol, containing 0.05% perfluoropentanoic acid (PFPA) and 0.1% formic acid (FA). Another aliquot was also analyzed using acidic positive ion conditions; however, it was chromatographically optimized for more hydrophobic compounds. In this method, the extract was gradient-eluted from the same aforementioned C18 column using methanol, acetonitrile, water, 0.05% PFPA, and 0.01% FA and was operated at an overall higher organic content. Another aliquot was analyzed using basic negative ion-optimized conditions using a separate dedicated C18 column. The basic extracts were gradient-eluted from the column using methanol and water, however with 6.5 mM ammonium bicarbonate at pH 8. The fourth aliquot was analyzed via negative ionization after elution from an HILIC column (Waters UPLC BEH Amide, 2.1 mm × 150 mm, 1.7 μm) using a gradient consisting of water and acetonitrile with 10 mM ammonium formate (pH 10.8). The MS analysis alternated between MS and data-dependent MS scans using dynamic exclusion. The scan range varied slightly between methods but covered 70 to 1000 mass-to-charge ratio (*m/z*). Raw data files are archived and extracted as described below.

### Data extraction and quality assurance

The raw MS data were extracted and loaded into the Metabolon Laboratory Information Management System and underwent quality control (QC) examination. Peaks were identified by Metabolon’s proprietary peak integration software.

### Compound identification

Metabolites were identified by comparison to an in-house library of purified standards that contain the retention time/index (RI), *m/z*, and chromatographic data (including MS/MS spectral data) from Metabolon. Compound identifications are based on three criteria: retention index within a narrow RI window, mass match to the library ±10 parts per million, and the match of MS/MS forward and reverse scores.

### Data normalization

For metabolomics measurements spanning multiple days, each metabolite was corrected in the same run-day blocks by adjusting the medians of each run-day block to one and normalizing each data point proportionately (block correction). When the metabolite level is below the instrument’s detection limit, the level is imputed with the minimal measured level of that metabolite across all samples. The abundance of each metabolite was subsequently normalized by probabilistic quotient normalization method, which accounts for an overall estimation on the most probable dilution factor (*34*). Data are subsequently log_2_-transformed. All data are reported in table S6.

### Metabolomics analysis

We applied Spearman correlation between the frozen time of our metabolomics samples and the metabolite abundance. We identified 12 metabolites that were significantly correlated to freezing time (table S1C). Nevertheless, removing these 12 metabolites did not affect any following analysis results. We flagged these metabolites as potentially confounded and left the data intact for others to analyze in the future. Differential metabolite abundance test were conducted with nonparametric Mann-Whitney *U* tests, followed by multiple hypothesis correction via the Benjamini-Hochberg procedure.

### Weighted DA score analysis

We implemented a novel DA score to incorporate the fold change of metabolites in a pathway. The weighted DA score is calculated by applying a nonparametric differential abundant test (here, Mann-Whitney *U* test followed by Benjamini-Hochberg multiple hypothesis correction) between two conditions (tumor and normal). Within each pathway, the weighted DA score is defined as the weighted mean of significant increased and decreased metabolites in a pathway

$$\mathrm{DA}=\frac{\left(w_{1}x_{1}+{\mathrm{wx}}_{2}+\cdots +w_{n}x_{n}\right)}{\left(w_{1}+w_{2}+\cdots +w_{n}\right)}$$

where *w*_1_ to *w_n_* are absolute values of log_2_ fold change of measured metabolites (1...*n*) in a pathway. *x* is an indicator function. If metabolite *i* is in a pathway with

1) Significant adjusted *P* value (*P*_adj_ < 0.05) and log_2_ fold change ≥ 0, *x*_i_ is 1,

2) Significant adjusted *P* value (*P*_adj_ < 0.05) and log_2_ fold change < 0, *x*_i_ is −1,

3) Adjusted *P* value is not significant (*P*_adj_ > 0.05), *x*_i_ is 0.

### Citrate and *cis*-aconitate abundance validation

To further confirm the metabolite abundance of citrate and *cis*-aconitate in 12 HCC tumors and 13 tumor-adjacent normal tissue samples, we measured the abundance of these two metabolites using GC-MS and LC-MS.

Tissue samples were snap-frozen in liquid nitrogen immediately after harvesting. Metabolites were extracted and analyzed by GC-MS and LC-MS. For metabolite extraction, mortar and pestle were cooled with liquid nitrogen, and frozen tissue was ground to a fine powder. Pulverized tissue powder was transferred to a screw-cap plastic vial, and an ice-cold extraction solvent (20 liter/mg; acetonitrile:methanol:water = 40:40:20) was added. Samples were vortexed for 30 s, snap-frozen in liquid nitrogen for 1 min, thawed on wet ice, and sonicated for 5 min in ice-cold water. The process was repeated three times. Samples were centrifuged at 20,000*g* for 20 min at 4°C. Supernatant (400 liters) was collected and dried in a vacuum evaporator (Genevac EZ-2 Elite).

For LC-MS, dried extracts were resuspended in 30 liters of 97:3 water:methanol containing 10 mM tributylamine and 15 mM acetic acid. Samples were vortexed, incubated on ice for 20 min, and clarified by centrifugation at 20,000*g* for 20 min at 4°C. LC-MS analysis used a Zorbax RRHD Extend-C18 column (150 mm × 2.1 mm, 1.8-m particle size, Agilent Technologies). Solvent A was 10 mM tributylamine, 15 mM acetic acid in 97:3 water:methanol, and solvent B was 10 mM tributylamine and 15 mM acetic acid in 3:97 water:methanol, prepared according to the manufacturer’s instructions (MassHunter Metabolomics dMRM Database and Method, Agilent Technologies). LC separation was coupled to a 6470 triple quadrupole mass spectrometer (Agilent Technologies) that was operated in dynamic MRM scan type and negative ionization mode. *cis*-aconitate was identified at a retention time of ~14.5 min with an MRM transition of *m/z* 173 to 129 (primary transition used for quantitation) and *m/z* 173 to 85.1 (confirmatory). Peaks representing *cis*-aconitate were normalized to internal standard [deuterated 2-hydroxyglutarate (2-HG)] peak area.

For GC-MS, dried metabolite extracts were resuspended in 50 liters of methoxyamine hydrochloride (40 mg/ml in pyridine) and incubated at 30°C for 90 min with agitation. Metabolites were further derivatized by the addition of 80 liters of *N*-methyl-*N*-(trimethylsilyl) trifluoroacetamide + 1% 2,2,2-trifluoro-*N*-methyl-*N*-(trimethylsilyl)-acetamide, chlorotrimethylsilane (Thermo Fisher Scientific) and 70 liters of ethyl acetate (Sigma-Aldrich) and incubated at 37°C for 30 min. Samples were diluted 1:2 with 200 liters of ethyl acetate and then analyzed using an Agilent 7890A GC coupled to Agilent 5977 mass spectrometer. The GC was operated in splitless mode with a constant helium carrier gas flow of 1 ml/min and with an HP-5ms column (Agilent Technologies). The injection volume was 1 liter, and the GC oven temperature was ramped from 60° to 290°C over 25 min. Peaks representing compounds of interest were extracted, integrated using MassHunter vB.08.00 (Agilent Technologies), and then normalized to internal standard (deuterated 2HG) peak area. Ions used for quantification of metabolite levels were citrate *m/z* 465 (confirmatory ion *m/z* 375) and deuterated 2HG *m/z* 252 (confirmatory ion *m/z* 354).

Compound identities were confirmed by injection of pure standards and sample spike-ins. To quantify concentrations of metabolites in tissue samples, standard curves for each metabolite were generated by running pure standards of known concentration on both GC-MS and LC-MS. Quantities of metabolites in tissue samples were calculated by plotting the normalized peak areas to standard curves of each respective metabolite and dividing by the mass of tissue used for metabolite extraction.

### Immunohistochemistry

Formalin-fixed paraffin-embedded tissue sections of tumors were sectioned onto glass slides at 4 μm in thickness, and consecutive tissue sections were stained with antibodies by the Molecular Cytology Core Facility at Memorial Sloan Kettering Cancer Center using Discovery XT processor (Ventana Medical Systems). Hematoxylin and eosin stains were performed under standard procedures and reviewed by two head and neck pathologists (B.X. and R.G.) to confirm the histological diagnosis and evaluate additional immunostaining. Serial unstained slides (4 μm) were prepared from each block for subsequent IHC with the following antibody clones: forkhead box P3 (FOXP3)(236A/E7) (Abcam, Waltham, MA), glutathione peroxidase 4 (EPNCIR144) (Abcam), PD-L1 (clone: E1L3N, Cell Signaling Technologies, Danvers, MA, USA; dilution, 1:400), PD-1 [clone: NAT105, ready to use (RTU), Cell Marque], CD4 (clone: SP35, Cell Maque; dilution, 1:12.5), CD8 (clone: SP57, RTU, Ventana Medical Systems, Tucson, AZ, USA), and CD68 (clone: KP1, RTU, Ventana Medical Systems, Tucson, AZ, USA). The sections were stained on the Ventana BenchMark ULTRA automated staining platform (Ventana Medical Systems, Tucson, AZ, USA) or on Leica Bond-III Autostainer (Leica Biosystems, Buffalo Grove, IL, USA), according to the manufacturer’s instructions. Each IHC stain was evaluated and qualified by a head and neck pathologist (B.X). For PD-L1, positive TC staining was defined as either partial or complete membranous staining of any intensity. Positive immune cell (IC) staining was defined as cytoplasmic or membranous staining of any intensity. Only tumor-infiltrating ICs were included in IC scoring. The combined positive score was defined as the number of PD-L1–positive TCs and ICs divided by a total number of TCs × 100. For all other IHCs, the number of ICs positive for each stain was counted manually at ×400 magnification (field diameter, 0.55 mm) at the hotspot. A hotspot is defined as the high power field with the highest density of positive ICs.

### Cluster-of-cluster assignments

The coca package was used to derive the consensus subtype calls of HCC tumors from RNA-seq (*n* = 53) and metabolomics platforms (*n* = 32), individually. In the first step, median absolute deviation (MAD) is used to select the top 3000 genes from RNA-seq data and the top 400 metabolites from the metabolome data. In the second step, a data matrix from a random sampling of 80% sample of submatrix after MAD selection was used to run hierarchical clustering with Euclidean distance and ward’s method. In the third step, the second step is repeated 50 times to obtain consensus subtype calls from RNA-seq and metabolomics data. The max concordance value was used to decide the best number of clusters *k* varying from *k* = 2 to *k* = 6. The best number of clusters from RNA-seq was *k* = 3, and the best number of clusters from metabolomics was *k* = 3. Although the number of clusters from RNA-seq and metabolomics both equal to 3, the clustering membership for each sample was not redundant across the two data modalities. There are 28 HCC tumors both having RNA-seq and metabolomics data. The subtype calls from individual platforms (RNA-seq and metabolomics) of these 28 samples were further encoded into a binary matrix as the input for consensus clustering procedure to identify the cluster-of-cluster assignments. Although *k* = 5 demonstrated the maximal concordance value in the cluster-of-cluster assignment, we selected *k* = 4 to enable each cluster to have a sufficiently large number of samples in it.

In the metabolite abundance heatmap of COCA cluster (Fig. 4A), we run differential metabolite abundance test (Wilcoxon rank sum test) to obtain the *P* value and log_2_ fold change for samples in a COCA cluster versus samples in other clusters. We use Benjamini-Hochberg method to obtain multiple hypotheses corrected *P* value. We only show significantly expressed metabolites in the heatmap (*q* < 0.05 and log_2_ fold change > 1 in a COCA cluster versus samples in other clusters). Likewise, in the gene expression heatmap of COCA cluster (fig. S4A), we run differential gene expression test (moderated *T* test) to obtain the *P* value and log_2_ fold change for samples in a COCA cluster versus samples in other clusters. We only show significantly expressed genes in the heatmap (*q* < 0.005 and log_2_ fold change > 1.5 in a COCA cluster versus samples in other clusters).

### Immune infiltration and immune activity analyses

Several orthogonal tools for assessing immune infiltration and activity in tumors using bulk RNA-seq data were applied. Cell-type Identification by Estimating Relative Subsets of RNA Transcripts (CIBERSORT) uses a reference gene expression signature and performs a linear support vector regression to adaptively select genes from the reference (*35*). ssGSEA (*23*) calculates enrichment scores for a sample and gene set pair, allowing clustering by pathways rather than individual genes, and generates metrics such as IIS and TIS as described by Şenbabaoğlu *et al.* (*36*). IIS is an aggregate score for innate and adaptive immune scores, while TIS is an aggregate score of nine T cell subtypes. Estimation of Stromal and Immune Cells in Malignant Tumor Tissues using Expression Data (ESTIMATE) is an ssGSEA-based technique, in which differential gene expression from high and low IC infiltrating tumor samples is used to derive a 141-gene signature estimating the degree of stromal and immune infiltration in a tumor (bioinformatics.mdanderson.org/estimate) (*25*). Immune cytolytic activity (“CYT” score) is calculated from geometric means of transcript levels of the two effector genes granzyme A and perforin 1.

## Notes

View/request a protocol for this paper from *Bio-protocol*.
