# Supplementary material for: Mitonuclear genotype remodels the metabolic and microenvironmental landscape of Hürthle cell carcinoma
Source: Sci Adv. 2022 Jun 22;8(25):eabn9699. doi: 10.1126/sciadv.abn9699 (PMC9216518; doi:10.1126/sciadv.abn9699)

Science Advances
AAAS

Supplementary Materials for

Mitonuclear genotype remodels the metabolic and microenvironmental landscape of Hürthle cell carcinoma

Ian Ganly *et al.*

Corresponding author: Ian Ganly, ganlyi@mskcc.org; Timothy A. Chan, chant2@ccf.org; Ed Reznik, reznike@mskcc.org

Sci. Adv. **8**, eabn9699 (2022)
DOI: 10.1126/sciadv.abn9699

The PDF file includes:

Figs. S1 to S7

Other Supplementary Material for this manuscript includes the following:

Tables S1 to S6

Supplementary figure legends

Fig. S1. Differential test in HCC tumor vs. normal. (**A**) Volcano plot of differential gene expression test in HCC tumor vs. normal. (**B**) Two-dimensional weighted differential abundance (DA) score plot. X-axis: DA score from metabolomics data, Y-axis: DA score from gene expression data. (**C**) Metabologram in the TCA cycle pathway. (**D**) Metabologram in the glycolysis pathway.

Fig S2. GPX4 expression in HCC tumors and adjacent normal tissues. (A) *GPX4* shows increased positivity by immunohistochemistry in HCC tumors relative to normal tissues (P-value: 8.58×10^{-9}, Wilcoxson rank-sum test) (*: $p < 0.05$, **: $p < 0.01$, ***: $p < 0.001$, N.S.: not significant). (B) Two representative images of HCC tumors demonstrating GPX4 positivity in tumor and normal regions.

Fig S3. Heteroplasmy levels of truncating variants across thyroid cancer subtypes. (A) All forms of thyroid cancer, including but not limited to HCC, demonstrate numerous instances of samples with near-homoplasmic truncating mtDNA mutations.

Fig. S4. Comparative metabolomics. (**A**) Volcano plot of differential abundance test in PDTC and TCV-PTC vs. normal. (**B**) $\log_2$ fold change in HCC/normal vs $\log_2$ fold change in HCC/PDTC, TCV-PTC. (**C**) Volcano plot of differential abundance test in all tumors (HCC, HA, PDTC, and TCV-PTC) vs. normal. (**D**) Log2 fold change of metabolite abundance between HCC tumor/normal and PDTC, TCV-PTC tumor/normal. (**E**) Significantly differentially abundant metabolites in HCC (red color) and other cancer types (black color).

Fig. S5. Significant TME features in HCC. (**A**) Correlation of tumor purity from ESTIMATE and from FACET approach.

Fig. S6. Significant gene expression features in COCA clustering and differential metabolite abundance test regards to different genetic alterations. (**A**) Heatmap of Significant gene expression features in COCA clustering. (**B**) Volcano plot of differential metabolite abundance tested with or without mtDNA mutations. (**C**) Volcano plot of differential metabolite abundance test in with or without gLOH. (**D**) Volcano plot of differential metabolite abundance tested with or without TERT mutations. (**E**) Volcano plot of differential metabolite abundance tested with or without mutations in the mTOR pathway.

Fig S7. Clinical and immunophenotypic correlates of HCC clusters. Integrative clusters show differences in (**A**) Progression-free survival and (**B**) Overall survival which do not reach statistical significance. Integrative clusters show no significant differences in expression signatures associated with either (**C**) M1 or (**D**) M2 macrophages. (**E**) *FOPX3* shows increased positivity by immunohistochemistry in HCC C4 tumors relative to other three COCA clusters (P-value: 0.21, Wilcoxon rank-sum test).

Supplementary Tables

Table S1

(**A**) HCC data summary. (**B**) Sample frozen time. (**C**) Spearman correlation between sample frozen time and metabolite abundance. Twelve metabolites significantly correlated with frozen time are marked in red color (q-value < 0.01). (**D**) Result of differential abundance test in all tumors vs. normals. (**E**) Result of differential abundance test in HCC HWIDE tumors vs. HCC HMIN tumors. (**F**) Result of differential abundance test in HCC tumor vs normal. (**G**) The concordance statistics between pyruvate/lactate ratio and metabolite abundance (**H**) name of metabolite in each of 85 KEGG metabolic pathways. (**I**) Result of weighted differential abundance score from metabolomics data.

Table S2

(**A**) Result of differential abundance test in HCC tumor vs HA. (**B**) Result of differential abundance test in PDTC and TCV-PTC vs normal. (**C**) Result of differential abundance test in HCC tumor vs PDTC and TCV-PTC.

Table S3

(**A**) TME features in from 28 HCC tumors with both RNA-seq and matched metabolomics data. (**B**) TME features in from 21 HCC tumors with RNA-seq but without matched metabolomics data.

Table S4

(**A**) Recurrence-free and overall survival time for 28 HCC tumors. (**B** to **E**) KEGG metabolic pathways from RNA GSEA analysis in each consensus cluster. (**F** to **I**), KEGG metabolic pathways from metabolite GSEA analysis in each consensus cluster.

Table S5

(**A** to **D),** Significant TME features in each COCA cluster v.s. others.

Table S6

(**A**) Log2-normalized metabolomics data.

Fig. S1. Differential test in HCC tumor vs. normal.

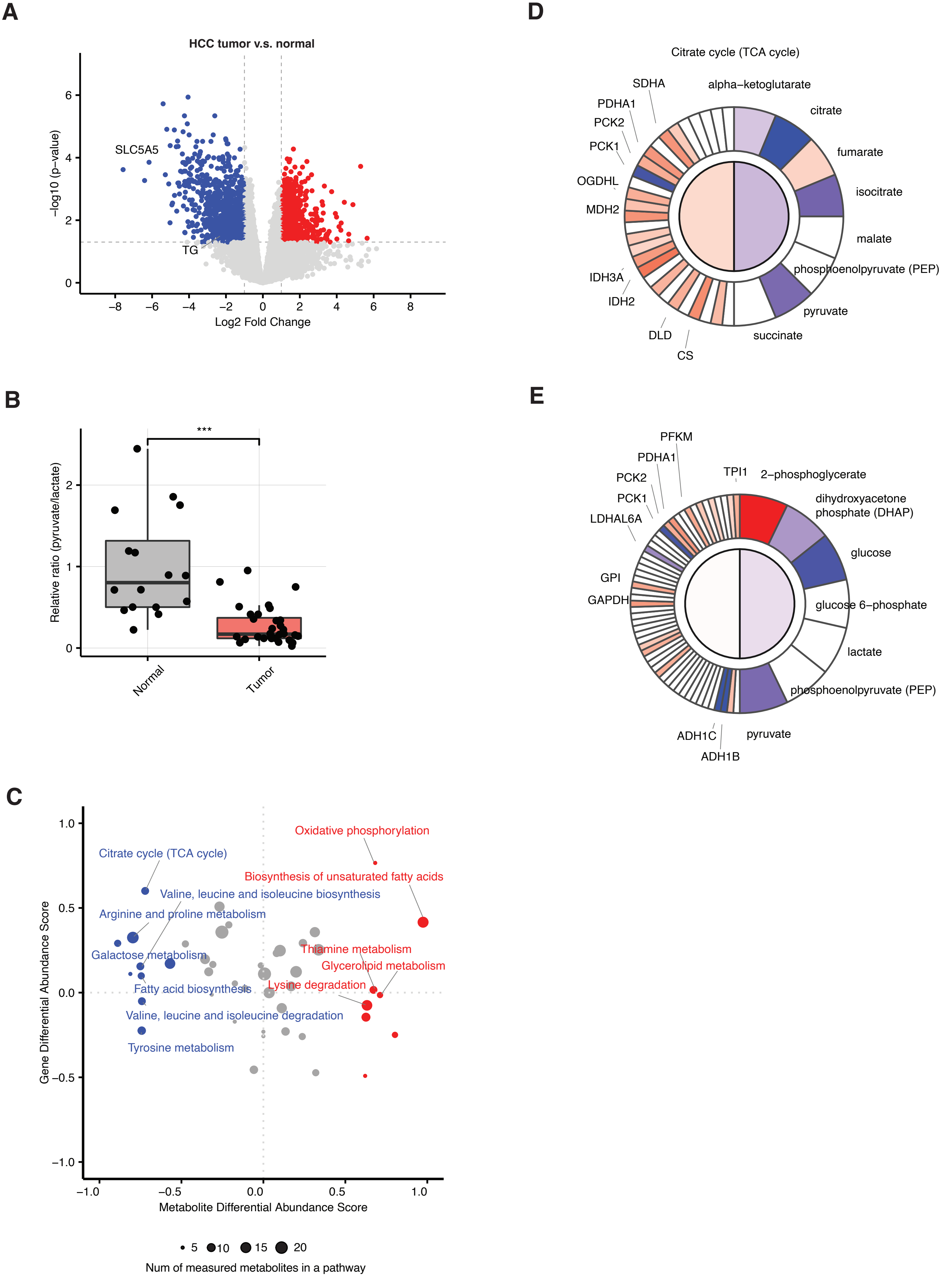

Fig S2. GPX4 expression in HCC tumors and adjacent normal tissues.

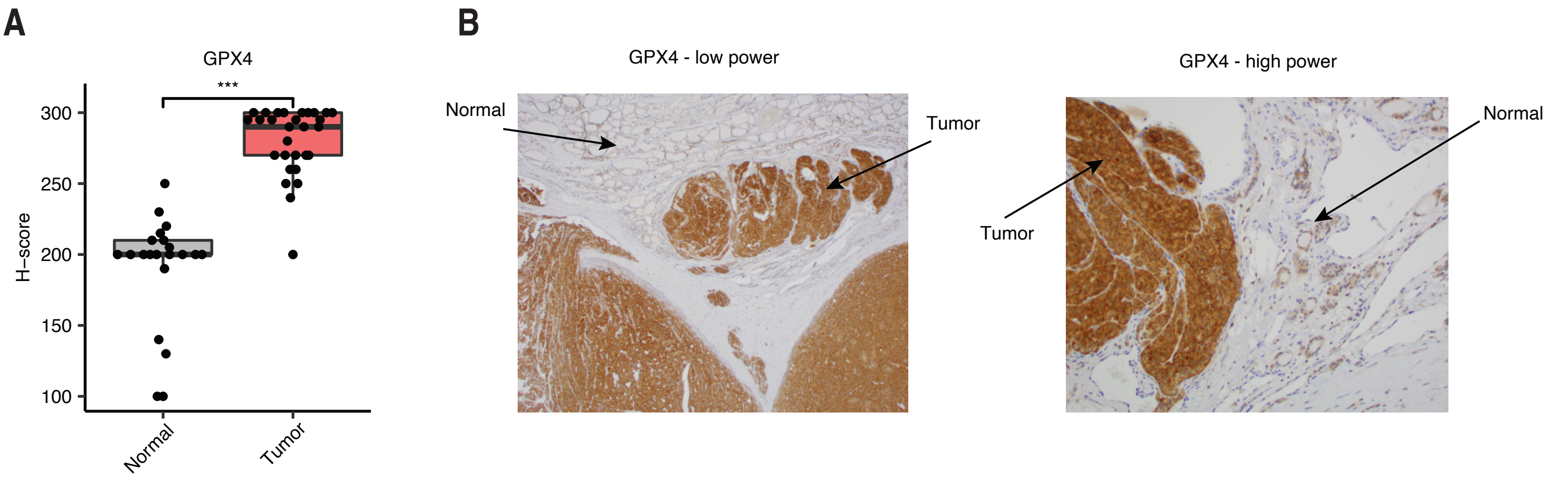

Fig S3. Heteroplasmy levels of truncating variants across thyroid cancer subtypes.

A

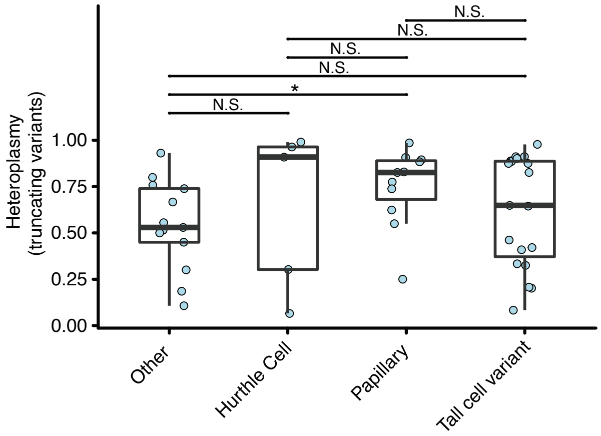

Fig. S4. Comparative metabolomics.

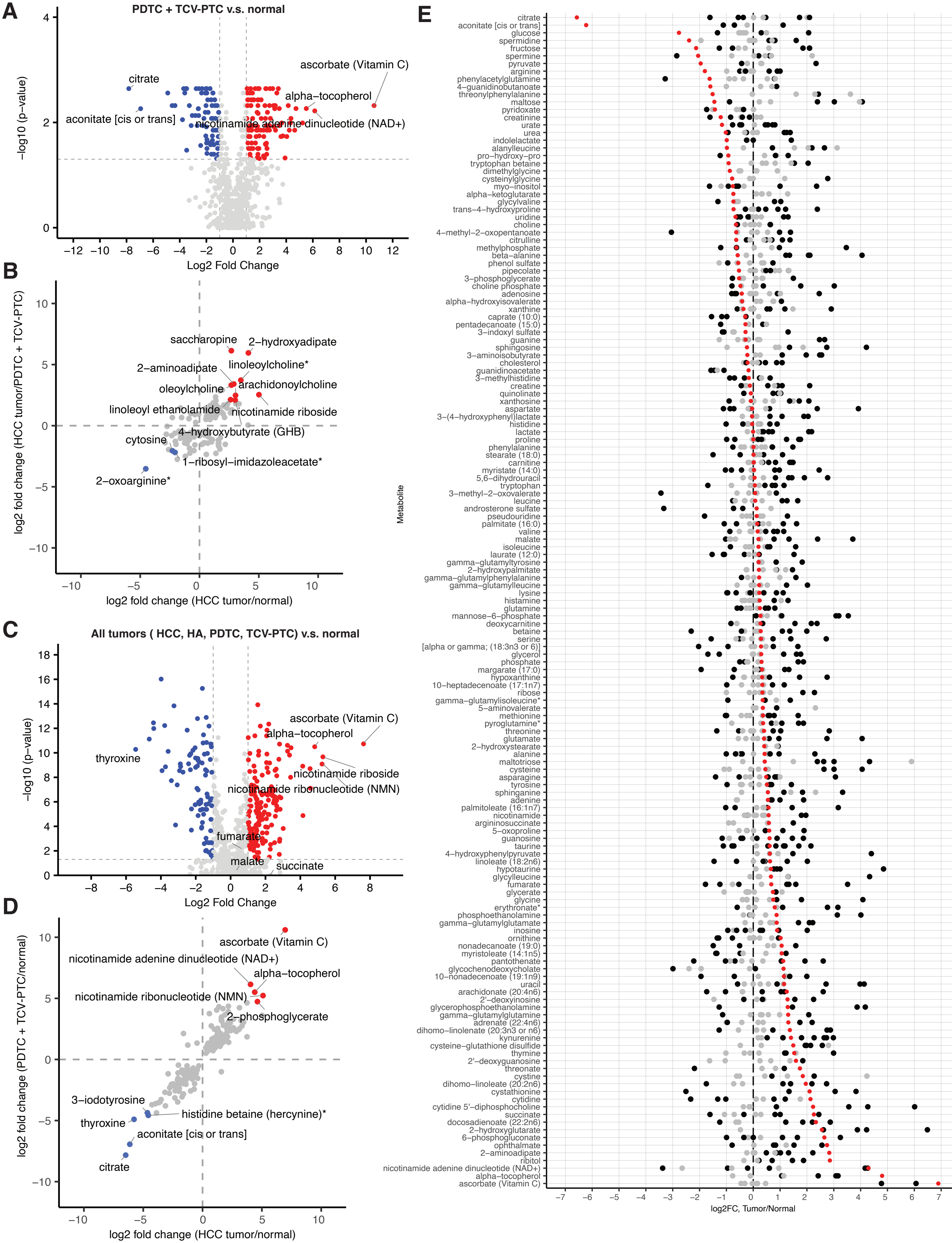

Fig. S5. Significant TME features in HCC.

A

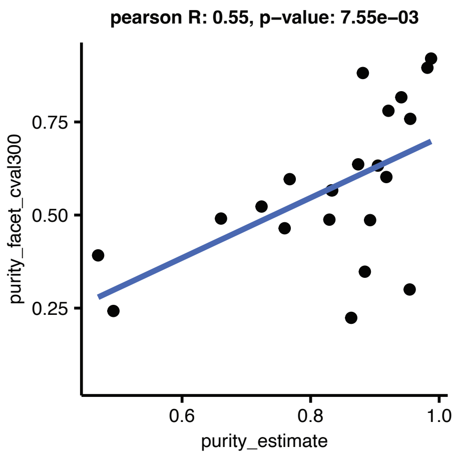

Fig. S6. Significant gene expression features in COCA clustering and differential metabolite abundance test regards to different genetic alterations.

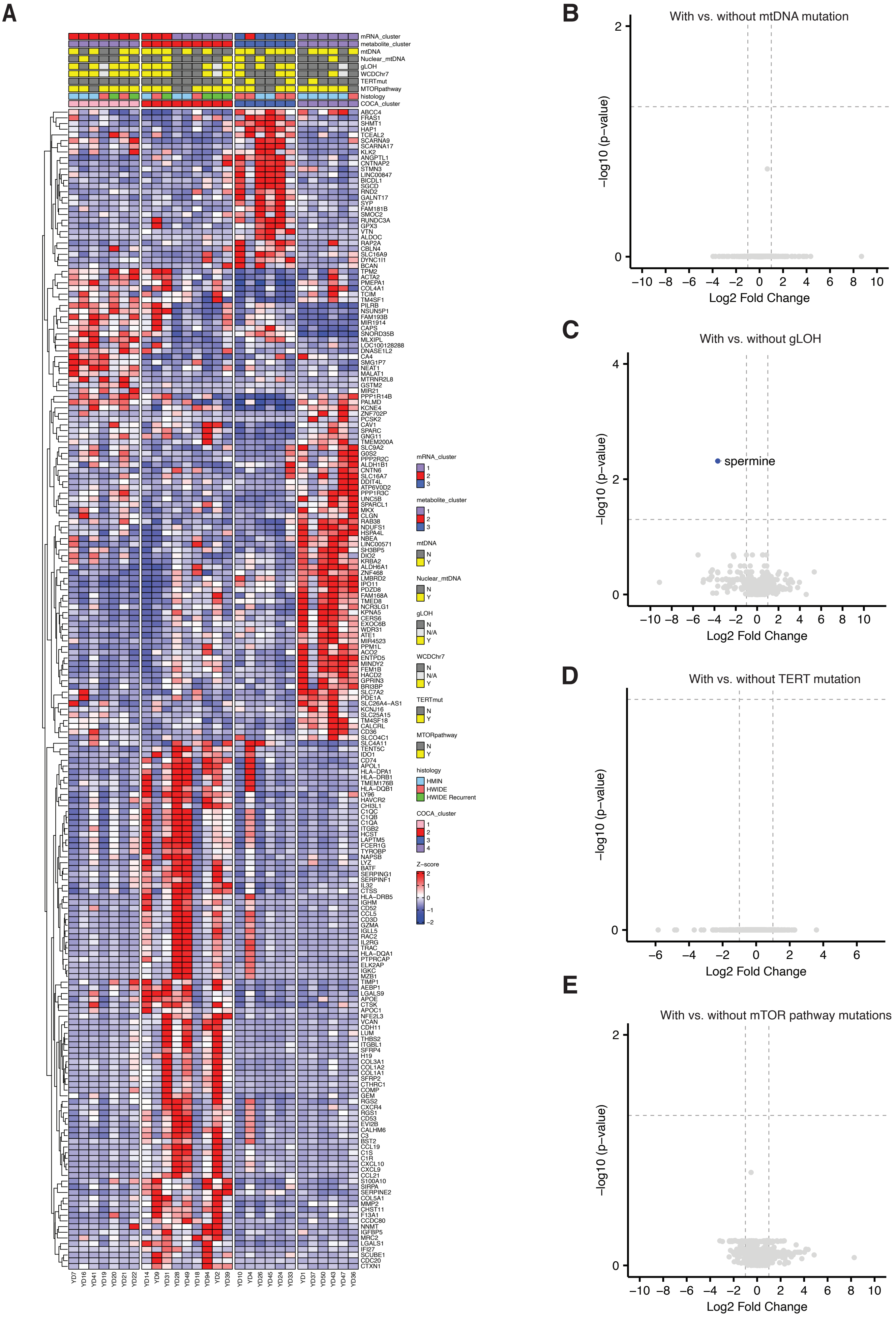

Fig S7. Clinical and immunophenotypic correlates of HCC clusters.

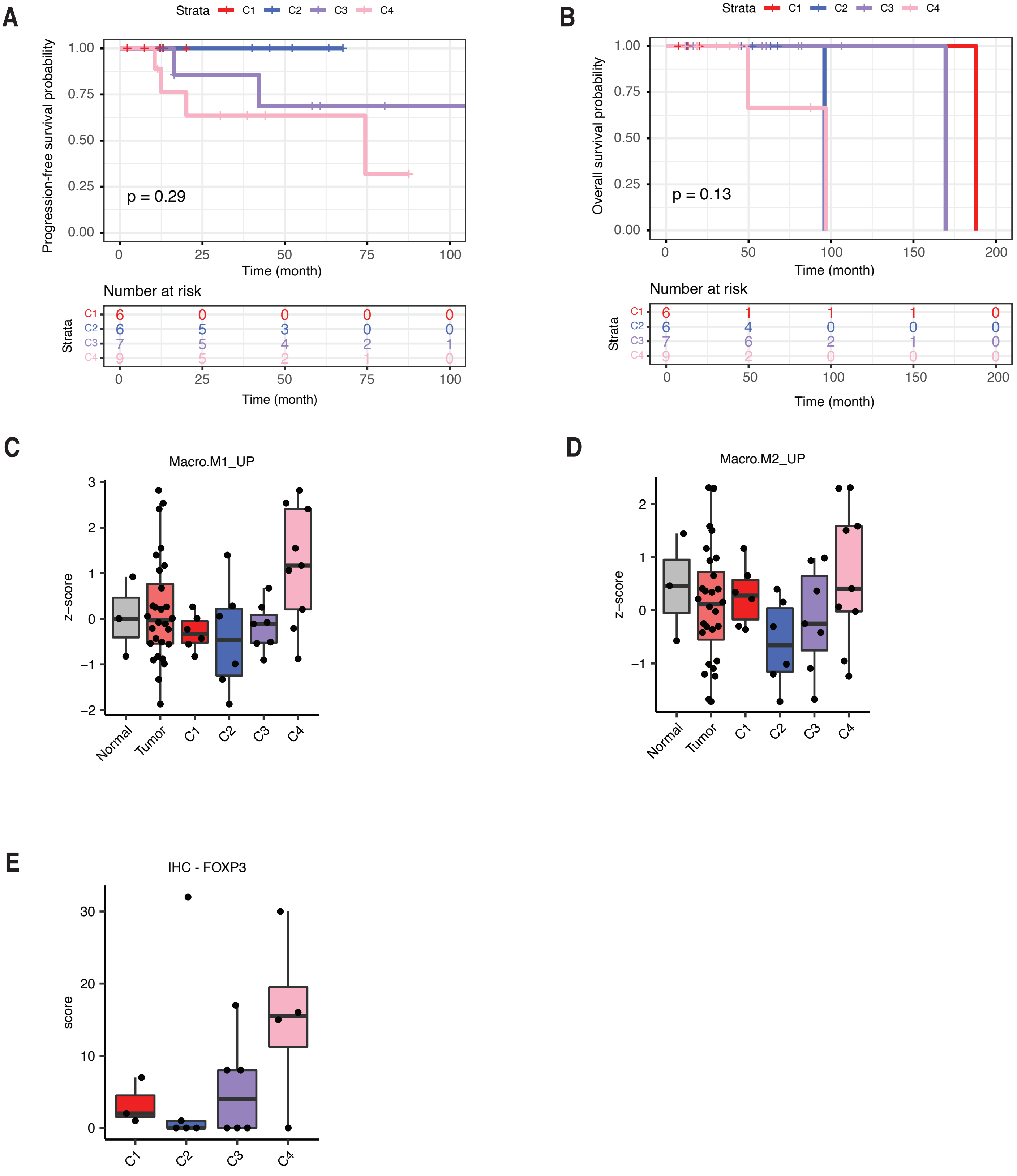

Supplement: Supplementary file 1 — Figs. S1 to S7 [file sciadv.abn9699_sm.pdf]
